# Exome sequencing for diagnosis of congenital hemolytic anemia

**DOI:** 10.1186/s13023-020-01425-5

**Published:** 2020-07-08

**Authors:** Lamisse Mansour-Hendili, Abdelrazak Aissat, Bouchra Badaoui, Mehdi Sakka, Christine Gameiro, Valérie Ortonne, Orianne Wagner-Ballon, Serge Pissard, Véronique Picard, Khaldoun Ghazal, Michel Bahuau, Corinne Guitton, Ziad Mansour, Mylène Duplan, Arnaud Petit, Nathalie Costedoat-Chalumeau, Marc Michel, Pablo Bartolucci, Stéphane Moutereau, Benoît Funalot, Frédéric Galactéros

**Affiliations:** 1 Département de Biochimie-Biologie Moléculaire, Pharmacologie, Génétique Médicale, AP-HP, Hôpitaux Universitaires Henri Mondor, F-94010 Creteil, France; 2 Univ Paris Est Creteil, INSERM, IMRB, F-94010 Creteil, France; 3 Département d’hématologie et d’immunologie, AP-HP, Hôpitaux Universitaires Henri Mondor, F-94010 Creteil, France; 4 Département d’hématologie, AP-HP, Hôpital Bicêtre, F-94270 Le Kremlin-Bicêtre, France; 5 Département de Biochimie, AP-HP, Hôpital Bicêtre, F-94270 Le Kremlin-Bicêtre, France; 6 Département d’hématologie pédiatrique, AP-HP, Hôpital Bicêtre, F-94270 Le Kremlin-Bicêtre, France; 7 Clinique ADASSA, Maternité, F-67000 Strasbourg, France; 8 Département d’onco-hématologie pédiatrique, CHU d’Angers, 4 Rue Larrey, 49100 Angers, France; 9 Département d’onco-hématologie pédiatrique, AP-HP, Hôpital Armand Trousseau, F-75012 Paris, France; 10 Département de médecine interne, AP-HP, Hôpital Cochin, F-75014 Paris, France; 11 Département de médecine interne, AP-HP, Hôpitaux Universitaires Henri Mondor, F-94010 Creteil, France; 12 Unité des maladies génétiques du globule rouge (UMGGR), AP-HP, Hôpitaux Universitaires Henri Mondor, F-94010 Creteil, France

**Keywords:** Hemolysis, Red blood cell, Membrane, NGS, Anemia, Congenital, Mutation

## Abstract

**Background:**

Congenital hemolytic anemia constitutes a heterogeneous group of rare genetic disorders of red blood cells. Diagnosis is based on clinical data, family history and phenotypic testing, genetic analyses being usually performed as a late step. In this study, we explored 40 patients with congenital hemolytic anemia by whole exome sequencing: 20 patients with hereditary spherocytosis and 20 patients with unexplained hemolysis.

**Results:**

A probable genetic cause of disease was identified in 82.5% of the patients (33/40): 100% of those with suspected hereditary spherocytosis (20/20) and 65% of those with unexplained hemolysis (13/20). We found that several patients carried genetic variations in more than one gene (3/20 in the hereditary spherocytosis group, 6/13 fully elucidated patients in the unexplained hemolysis group), giving a more accurate picture of the genetic complexity of congenital hemolytic anemia. In addition, whole exome sequencing allowed us to identify genetic variants in non-congenital hemolytic anemia genes that explained part of the phenotype in 3 patients.

**Conclusion:**

The rapid development of next generation sequencing has rendered the genetic study of these diseases much easier and cheaper. Whole exome sequencing in congenital hemolytic anemia could provide a more precise and quicker diagnosis, improve patients’ healthcare and probably has to be democratized notably for complex cases.

## Background

Congenital hemolytic anemia (CHA) is a group of rare genetic disorders characterized by increased destruction of red blood cells (RBC) [1]. They result from corpuscular causes such as hemoglobin disorders, membrane diseases, RBC enzyme deficiencies or congenital dyserythropoietic anemia (CDA), or from extra-corpuscular causes such as atypical hemolytic and uremic syndromes (aHUS) due to complement pathways dysfunctions.

Membrane disorders [2–5] include hereditary spherocytosis (HS, the most frequent cause in European population with nearly 1/2000 subjects affected, MIM 182900, 182,870, 270,970, 612,653, 612,690) [6]; hereditary elliptocytosis (HE, from 1/5000–1/10,000 in European population to 1/100 in West African populations, MIM 130600, 109,270, 611,804) [7]; hereditary pyropoïkilocytosis (HPP, MIM 266140), which is a rare and severe subtype of HE, and hereditary stomatocytosis, with a dehydrated form (incidence of 1/50,000 individuals, MIM 616689) and an overhydrated form (incidence lower than 1/100,000, MIM 185000) [8]. Transmission is often autosomal dominant (AD) but de novo mutations or autosomal recessive (AR) forms (as is the case in HPP) can occur.

Defaults in enzymatic pathways of RBC metabolism are numerous [9] the most frequent being Glucose-6 Phosphate Dehydrogenase (G6PD, MIM 300908) and Pyruvate Kinase deficiencies (MIM 266200) [10]. Most of them have AR inheritance but some are X-Linked (XL) (as is the case for G6PD deficiency).

CDA (MIMs 224,120, 615,631, 224,100, 105,600, 613,673) is characterized by ineffective central erythropoiesis with progressive secondary iron overload and has AR, AD or XL transmission [11, 12]. Peripheral hemolysis can occur and misdiagnosis is often due to phenotypic overlap with other types of CHA [13, 14].

Clinical features of CHA vary from severe neonatal or even prenatal anemia with high morbidity and transfusion dependence to well-compensated hemolysis without anemia. In addition, clinical manifestations may be increased by associated comorbidities (such as hemochromatosis or hemoglobin variant) or during acute diseases, inflammatory conditions, or pregnancy.

Mutations causing CHA have been identified in more than 100 different genes, some of which are very large. Next generation sequencing (NGS) allows massive parallel sequencing of numerous genes and therefore appears as a very suitable approach for genetic dissection of CHA. Different NGS strategies have been developed over the last few years for CHA (Additional file 1, Supplementary Table S1). They included targeted gene panels (comprising 28 to 76 genes) with a success rate for diagnosis varying from 38 to 90%, depending on proband numbers (ranging from 2 to 62) and subtypes of anemia [15–23], and whole-exome sequencing (WES) in a few studies (limited to 1 to 7 probands, except two studies focusing on 38 Chinese cases of HS [24] and 24 cases of autosomal recessive HS [25]), with success rates between 29 and 100% [14, 24–32]. We used WES to explore 40 CHA patients: 20 with suspected HS and 20 with unexplained hemolysis (UH) despite available phenotype exploration. We first analyzed genomic data using a predefined list of 71 CHA genes (Additional file 1, Supplementary Table 2) and extended the analysis to WES when necessary. This approach allowed us to find a probable molecular cause in 82.5% of patients.

## Methods (see supplementary material for more details)

### Biochemical and hematologic tests

Standard laboratory tests included cell blood count, blood smear examination, hemolysis markers, RBC density (measured by the phthalate density-distribution method, as described by Bartolucci et al in 2012 [33]**,** with density curves profiles), hemoglobin study (beginning with isoelectric focusing and HPLC. In case of abnormalities, acid agar gel electrophoresis, Itano test, capillary electrophoresis and reverse phase HPLC could be performed, RBC enzymes activity (G6PD, Pyruvate Kinase, Hexokinase routinely measured) and EMA test **(**performed following the methods described by King et al in 2000 [34] and Girodon et al in 2008 [35]). When possible, osmolar gradient ektacytometry (Technicon®) and membrane protein electrophoresis (MPE) were performed.

#### DNA extraction

Peripheral blood EDTA sample was obtained from patients after written informed consent for genetic analysis. Genomic DNA was extracted using the Flexigene extraction kit, following manufacturer’s recommendations (Qiagen; Hilden, Germany).

#### Sequencing and data analysis

**Library preparation** was performed from 100 ng of enzymatically fragmented genomic DNA using the Kapa library hyperprep kit, following manufacturer’s recommendations (KAPABIOSYSTEMS, Wilmington, Massachusetts). Enrichment for exonic sequences was performed using the Medexome kit, following manufacturer’s recommendations (NimbleGen, Madison, Wisconsin).

### NGS sequencing

Captured libraries were sequenced on a Nextseq500 instrument (Illumina, San Diego, California, USA) with high-output FlowCell and reagents, in order to obtain 150 bp paired-end reads.

### Bioinformatic analyses

Demultiplexing and .fastq files generation were performed using Bcl2fastq software (v.1.8.4). Trimming of low-quality bases (Phred score < 10) and adapter-contaminated ends was performed with Trimmomatic (v.0.6.1) (http://www.bioinformatics.babraham.ac.uk/projects/trim_galore/). Alignment and variant calling were performed in duplicates, using both an in-house pipeline following the BWA/GATK best practices and the Seqnext software (JSI, Ettenheim, Germany). For our in-house bioinformatic pipeline, high-quality reads were mapped on the Human reference genome (GRCh37, hg19) using BWA-MEM (v.0.7.1). SAMtools (http://www.htslib.org/) and Picard (v.1.106, https://broadinstitute.github.io/picard/) were run to remove the duplicate reads. Mean depth of coverage was >171x for all samples with > 97.6% covered at least 30X. Targeted regions with depth under 30X were selected with the Depth Of Coverage GATK tool and manually checked using bam files loaded on two reads viewers, i.e. Alamut Visual (Interactive Biosoftware, Rouen, France) and IGV (Broad institute, Cambridge, MA 02142, USA). For our first-line list of genes, all targeted regions were above 30X, excepted *GPX1 and PGD* first exons, which were sequenced by Sanger. Exon 1 of *PIEZO1* was usually covered ~15X but was validated after a manual check of high-quality reads on bam viewers.

Variant calling was performed using the Genome Analysis Toolkit (v.3.8) with the Haplotype Caller tool in GVCF mode. VQSR filters were applied according to GATK guidelines (when QD < 2, FS > 60 for snps and > 200 for indels, ReadPosRankSum <− 8 for snps and > − 20 for indels, when MQ < 40, MQRankSum <− 12.5 and SOR < 3).

### Variant annotation

The quality-filtered variant calling files (VCF) obtained were gene- and region-based annotated on GRCh37, hg19 reference genome using Annovar [36] and dbSNP141.

Functional annotation was made using in silico prediction tools and amino acid conservation scores (SIFT (http://sift.jcvi.org/), PolyPhen-2 (http://genetics.bwh.harvard.edu/pph2/), Mutation Taster (http://www.mutationtaster.org/), align GVGD (http://agvgd.iarc.fr/) and CADD (https://cadd.gs.washington.edu/)). MaxEntScan (http://genes.mit.edu/burgelab/maxent/Xmaxentscan_scoreseq.html) and Human Splicing Finder3.0 (http://www.umd.be/HSF/) were used for splicing effect predictions. Protein domain functions were determined by using the SMART software (http://smart.embl-heidelberg.de/) and are available in Table S3. Functional classification following ACMG criteria [37] was performed using InterVar [38] and Ingenuity Variant Analysis (IVA) software (Qiagen).

### Variants filtering and selection

According to ACMG guidelines, we excluded all variants tagged as Benign.

According to Annovar gene- or region-based annotations, we excluded variants disrupting microRNA binding sites, promoter, intergenic, ncRNA as well as intronic variants > 10 nucleotides of a splice site (−splicing_threshold 10) and kept only those present on an alternative transcript. Variants in regions with segmental duplications were excluded (genomicSuperDups).

All the possible inheritance patterns were tested according to disease knowledge. Variants with population frequency (Minor Allele Frequency, MAF) in gnomAD, ExAC, 1000 g and ESP6500 higher than 5% for AR disorders and higher than 1% for AD disorders were filtered out, except variants known to have modulating effects on phenotype, such as *HFE* variants (H63D or C282Y) involved in iron overload and the *SPTA1* alpha-Lely polymorphism (c.6531-12C > T) involved in membrane disorders. This latter variant is known to be pathogenic when associated in *trans* with a deleterious variant of *SPTA1* [39].

The first-line analysis was focused on 71 known CHA genes as a target gene panel (Additionnal file 1, Supplementary Table S2). An extended WES analysis was performed using IVA filters “biological context” and “phenotype driven”. In addition, we performed a complementary analysis of 2138 genes encoding erythrocyte proteins identified by proteomic studies [40–42].

### New variants

All new variants classified as VUS, likely pathogenic or pathogenic have been registered in the Clinvar database (https://www-ncbi-nlm-nih-gov.gate2.inist.fr/clinvar/ see Additional file 1, Supplementary Table S4).

### Sanger sequencing

Sanger sequencing was used to confirm each potentially deleterious variation found by NGS. Primers sequences and PCR conditions are available upon request.

#### Patients

Forty patients suffering from non-immune hemolysis with negative direct antiglobulin tests were submitted to WES sequencing. Clinical and biological data are available in Supplementary Tables S5 and S6 (normal values in Additional file 1 and Supplementary Table S7). Blood smear results and genetic results are available in Tables S8 and S11. In silico analysis of genetic variants is available in Additional file 1, supplementary Table S3.

Twenty patients had a HS phenotype, among which four were apparently sporadic cases (**P1, P3, P13, P14**). The 20 other patients had UH, with either discordant or non-conclusive phenotypical tests. For several patients, EMA test results were discordant with ektacytometry. In the other cases, EMA test and ektacytometry were either normal or atypical. Three of these patients had UH associated to a sickle cell trait (A/S). Several patients had no familial history. Despite of this fact they were considered as potential cases of CHA because phenotypic exploration of common acquired causes of hemolytic anemia such as complete antiglobulin tests (Coombs test) and paroxysmal nocturnal hemoglobinuria test remained negative.

## Results

Genetic results are available in Tables 1 and 2 and in Additional file 1, supplementary Tables S3 and S8 to S11.Clinico-biological data are available in Additional file 1, supplementary Tables S5 to S7.

**Table 1:** Genetic results of HS patient

Patient ID	Gene name	transcript	Nucleotide change	AA change	zygosity	References rsNumber	gnomAD allele frequency	Polyphen-2	Mutation taster	MaxEnt Scan	CADD score	ACMG class
P1	*ANK1*	NM_020476	c.5152C > T	p.Gln1718*	het	No	0	NA	NA		37	LP
	*HBA1*	NM_0005558	c.389 T > C	p.L130P	het	Darbellay et al 1995	0	PD	DC		23.4	LP
P2	*ANK1*	NM_020476	c.1702-2A > C		het	No ref	0	NA	NA	−100%	34	LP
	*HFE*	NM_000410	c.187C > G	p.H63D	het	Kaczorowska-Hac et al 2016 rs1799945	10.83%	B	P		12.8	VUS
	*HFE*	NM_000410	c.845G > A	p.C282Y	het	Kaczorowska-Hac et al 2016 rs1800562	3.37%	PD	DC		25.2	P
P3	*SLC4A1*	NM_000342	c.1458C > G	p.Y486*	het	No	0	NA	NA		35	LP
P4	*SLC4A1*	NM_000342	c.486-2A > G		het	no	0	NA	NA	−100%	25.3	P
P5	*SPTB*	NM_001024858	c.1331_1338del	p.Leu444Profs*3	het	Dhermy et al 1998	0	NA	DC			P
P6	*ANK1*	NM_020476	c.5497C > T	p.R1833*	het	Hayette et al 1998	0				47	P
*P7*	*SLC4A1*	NM_000342	c.1322 T > G	p.L441R	*het*	No	*0*	PD	DC	*No effect*	26.8	VUS
*P8*	*ANK1*	NM_020476	c.1801-17G > A		*het*	Duru et al rs786205243	*0*			Creation of a cryptic acceptor	8.2	LP
*P9*	*ANK1*	NM_020476	c.4462C > T	p.R1488*	het	Ozcan et al 2003 rs777701149	*0*				36	P
*P10*	*ANK1*	NM_020476	c.1A > G	p.?	het	no	0	NA	NA	NA	14.3	P
*P11*	*SPTB*	NM_001024858	c.2863C > T	p.R955*	het	no	0	NA	NA	NA	37	P
	*SPTA1*	NM_003126	c.6421C > T	p.R2141W	het	Niss et al 2016 rs41273519	0.2%	PD	DC		27.4	LP
*P12*	*SPTB*	NM_001024858	c.4973 + 5G > A		het	no	0	NA	NA	−100%	16.48	VUS
*P13*	*ANK1*	NM_020476	c.534delC	p.H178Qfs*75	het	no	0	NA	NA			LP
*P14*	*SPTB*	NM_001024858	c.5623C > T	p.Q1875*	het	no	0	NA	NA		48	LP
*P15*	*SLC4A1*	NM_000342	c.1462G > A	p.V488M	het	Alloisio et al 1997 rs28931584	0.00041%	PD	DC	No effect	24.5	P
*P16*	*ANK1*	NM_020476	c.712-2A > G		het	no	0	NA	NA	−100%	34	LP
*P17*	*SLC4A1*	NM_000342	c.2423G > A	p.R808H	het	Bogardus et al 2012 rs866727908	0	PD	DC	No effect	33	LP
	*PIEZO1*		c.2578G > A	p.V860M	het	rs532390680	0.0028%	PossD	DC	No effect		VUS
*P18*	*SLC4A1*	NM_000342	c.2279G > A	p.R760Q	het	Jarolim et al 1995	0	PD	DC	No effect	29.6	LP
*P19*	*SPTB*	NM_001024858	c.3436dup	p.L1146Pfs*36	het	no	0	NA	NA			LP
	*SPTB*	NM_001024858	c.6101G > A	p.S2034N	het	no	0.00041%	B	DC	No effect	22.9	VUS
*P20*	*SPTB*	NM_001024858	c.3916C > T	p.R1306*	het	No rs150471537	0	NA	NA		38	LP

Table 1 legend: Variants description and classification according to ACMG guidelines as benign likely benign (LB) variant of uncertain significance (VUS) likely pathogenic (LP) or pathogenic (P). In silico study of missense variations was assessed thanks to Polyphen-2, Mutation taster and CADD score algorithm. HGMD professional and pubmed web interface were used to check for variants description in litterature. Abbreviations: het: heterozygous state; hom: homozygous state; hem: hemizygous state; F: female; M: male; HS: hereditary spherocytosis; gnomAD: genome agregation database https://gnomad.broadinstitute.org; ND: not done; NA: not applicable; DC: disease causing; P: polymorphism; PD: probably damaging; PossD: possibly damaging; B: benign.

**Table 2:** Genetic results of UH patients

Patient ID	Gene name	Transcript	Nucleotide change	AA change	zygosity	references	gnomAD allele frequency	Polyphen-2	Mutation taster	MaxEnt Scan	CADD score	ACMG class
P21	*SPTA1*	NM_003126	c.6600 + 5G > T		het	no	0	NA	NA	−62.5%	18.74	VUS
	*SPTA1*	NM_003126	c.6531-12C > T		het	Alpha-Lely polymorphism rs28525570	25%					
P22	*SPTA1*	NM_003126	C.2898G > A	p.(=)	het	no	0	NA	NA	−29.3%	14.2	VUS
	*SPTA1*	NM_003126	c.6531-12C > T		het	Alpha-Lely polymorphism rs28525570	25%					
P23	*ALAS2*	NM_000032	c.-258C > G		het	Bekri et al 2003 rs140772352	0.54%	NA	NA	NA		VUS
P24	*TRPV4*	NM_021625	c.1913C > T	p.P638L	hom	No rs35058636	0.03% (no homozygotes)	B	DC	No effect		VUS
	*ADAR*	NM_001111	c.1586C > T	p.P529L	het	no	0	PD	DC	No effect		VUS
P25	*SEC23B*	NM_001172745	c.40C > T	p.R14W	het	Russo et al 2011 rs121918222	0.022%	PossD	DC	No effect		P
	*SEC23B*	NM_001172745	c.325G > A	p.E109K	het	Russo et al 2011 Rs121918221	0.023%	PD	DC	No effect		P
*P26*	*HAMP*	NM_021175	c.49_54del	p.L17_L18del	het	no	0	NA	NA	No effect		VUS
	*HFE*	NM_000410	c.845G > A	p.C282Y	het	rs1800562	3.37%	PD	P	No effect		P
	*CD46*	NM_172359	c.402 T > G	p.I134M	het	no	0	PossD	P	No effect		VUS
*P27*	*CFH*	NM_00186	c.2850G > T	p.Q950H	het	*Mohlin* et al 2015 rs149474608	0.39%	B	P	No effect		LB
*P28*	*SEC23B*	NM_001172745	c.1276G > A	p.V426I	het	Schwartz et al 2009 rs41309927	4.3%	B	P	No effect		VUS
	*CDAN1*	NM_138477	c.256C > T	p.P86S	het	No rs543791953	0.052%	B	P	No effect		VUS
*P29*	SPTA1	NM_003126	c.1688G > A	p.R563Q	het	No rs202243588	0.11%	PD	DC	Possible new acceptor site	25.1	VUS
	*HBB*	NM_000518	c.20A > T	p.E7V	het	Yes coding for HbS rs334	0.44%	B	P		13.8	P
	SPTA1	NM_003126	c.6531-12C > T		het	Alpha-Lely polymorphism rs28525570						
*P30*	*PIEZO1*	NM_001142864	c.1126C > G	p.P376A	het	no	0	B	P	Possible new acceptor site		VUS
*P31*	*KCNN4*	NM_002250	c.1055G > A	p.R352H	het	Rappetti Mauss et al 2015 rs774455945	0	PossD	DC	No effect		P
	*PIEZO1*	NM_001142864	c.3629C > T	p.A1210V	het	No rs761971227	0.006%	B	DC	No effect		LP
	*PIEZO1*	NM_001142864	c.3629C > T	p.A1210V	absence							
*P32*	*SPTB*	NM_001024858	c.[6706C > A;6737C > T]	p.[L2236M;A2246V]	Het in cis	no	0	B/PD	DC/P	No effect		VUS
	*HBB*	NM_000518	c.20A > T	p.E7V	het	Yes coding for HbS rs334	0.44%	B	P		13.8	P
*P33*	*G6PD*	NM_000402	c.538G > A	p.V180I	het	no	0	PossD	DC	No effect		VUS
	*SPTB*	NM_001024858	c.6271C > A	p.P2091T	het	No rs372733273	0.0065%	B	DC	no effect		VUS
*P34*	*HFE*	NM_000410	c.187C > G	p.H63D	hom	Kaczoeowska-Hac et al 2016 rs1799945	10.83%	B	P	No effect		LB
	*ABCG8*	NM_022437	c.-27G > A		het	no	0	NA	NA	NA		VUS
	*ADAMTS13*	NM_139025	c.119C > G	p.A40G	het	No rs782213090	0.00041%	B	P			VUS
	*ADAMTS13*	NM_139025	c.4007G > A	p.R1336Q	het	No No rs	0.0012%	PD	P			VUS
*P35*	*SH2B3*	NM_005475	c.1A > G	p.0?	het	no	0	NA	NA	No effect		LP
	*SCN9A*	NM_002977	c.2938G > T	p.A980S	het	no	0	PossD	DC	No effect		VUS
*P36*	*SPTA1*	NM_003126	c.6672A > C	p.E2224D	hom	No Rs142775522	1.5% no homozygotes	PD	DC	No effect	22.3	VUS
	*SLC4A1*	NM_000342	c.1199_1225del	p.A400_A408del	het	Wilder et al 2009 rs769664228	0.0047%	PD	DC			LP
	*PIEZO1*	NM_001142864	c.1369C > T	p.R457C	het	Russo et al 2018	0	PD	DC	No effect		LP
	*G6PD*	NM_000402	c.292G > A	p.V98M	het	Vulliamy et al 1988 rs1050828	1.15%					LP
	*HBB*	NM_000518	c.20A > T	p.E7V	het	Yes coding for HbS rs334	0.44%	B	P		13.8	P
*P37*	*SPTA1*	NM_003126	c.3291G > A	p.W1097*	het	no	0	NA	NA	NA	42	LP
	*SPTA1*	NM_003126	c.6531-12C > T		het	Alpha-Lely polymorphism rs28525570						
	*HFE*	NM_000410	c.187C > G	p.H63D	hom	Kaczoeowska-Hac et al 2016 rs1799945	10.83%	B	P	No effect		LB
*P38*	*CFH*	NM_00186	c.157C > T	p.R53C	het	Servais et al 2012 rs757785149	0.0014%	PD	DC	No effect		LP
	*PIEZO1*	NM_001142864	c.4246G > A	p.G1416R	het	No rs771605269	0.00033%	PD	DC	New cryptic acceptor site		VUS
*P39*	*SPTA1*	NM_003126	c.779 T > C	p.L260P	het	Marchesi et al 1987 Rs121918634	0.017% (Afr)	PD	DC	No effect		LP
	*SPTA1*	NM_003126	c.6531-12C > T		het	Alpha-Lely polymorphism rs28525570						
*P40*	*ATP11C*	NM_173694	c.2434C > T	p.P812S	hem	no	0.00055% no hemizygous	PD	DC	No effect		VUS
	*ANK1*	NM_020476	c.4558G > C	p.E1520Q	het	no	0.0021%	B	DC	No effect	26	VUS

Table 2 Legend: Variants description and classification according to ACMG guidelines as benign likely benign (LB), variant of uncertain significance. (VUS), likely pathogenic (LP) or pathogenic (P). In silico study of missense variations was assessed thanks to Polyphen-2, Mutation taster andCADD score algorithm. HGMD professional and pubmed web interface were used to check for variants description in litterature. Abbreviations: het: heterozygous state; hom: homozygous state; hem: hemizygous state; F: female; M: male; HS: hereditary spherocytosis; gnomAD: genome agregation database https://gnomad.broadinstitute.org; ND: not done; NA: not applicable; DC: disease causing; P: polymorphism; PD: probably damaging; PossD: possibly damaging; B: benign.

**Fig. 1:**
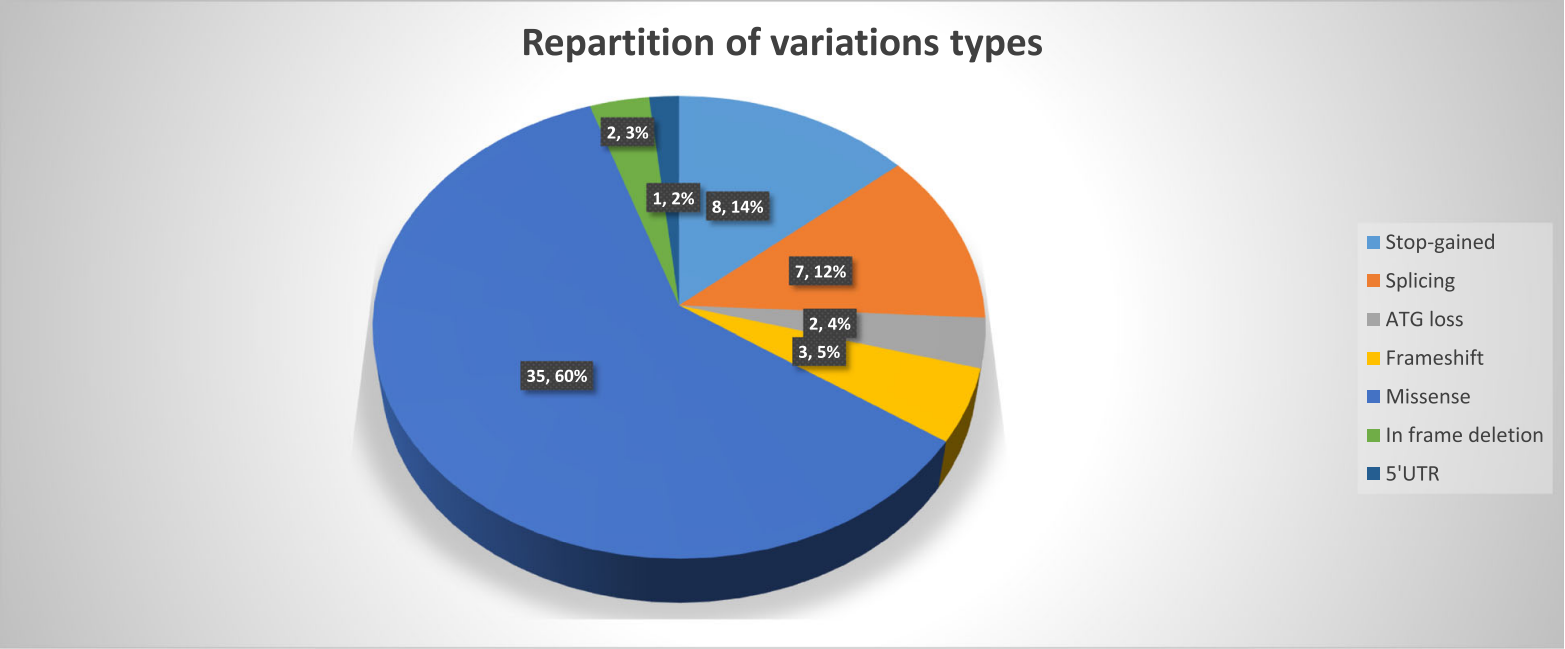
Distribution of mutations types

We defined two groups: one with clear phenotype (HS group) and one with unclear phenotype (UH group). The different types of variants identified are summarized in Fig. 1.

### HS group

**Fig. 2:**
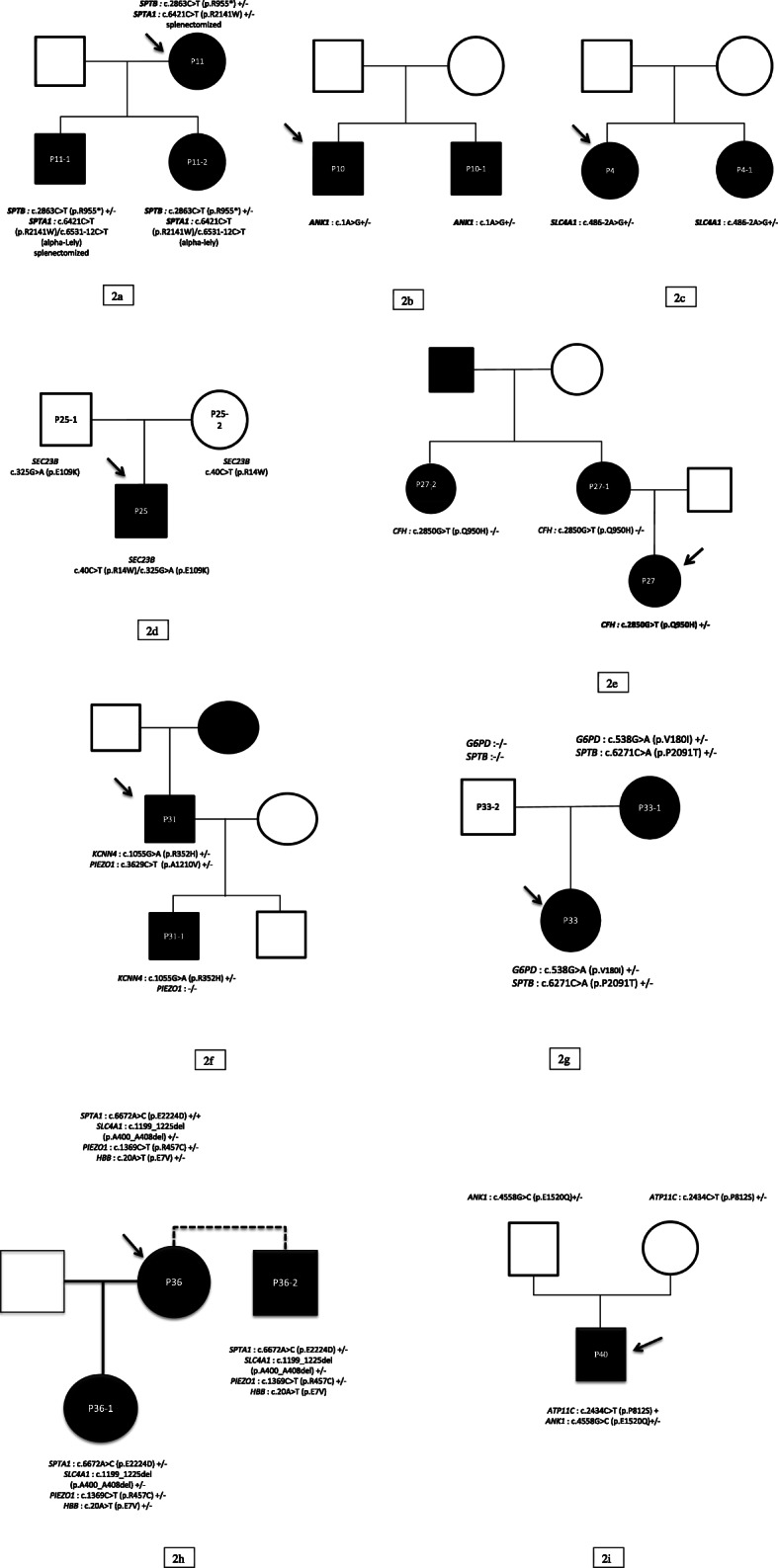
Pedigrees of 3 HS families and 6 UH families. Legend: Black squares: affected males; black circles: affected females; open squares: unaffected males; open circles: unaffected females; arrow: the proband. 2a = **P11** family pedigree (HS); 2b = **P10** family pedigree (HS); 2c = **P4** family pedigree (HS); 2d = **P25** family pedigree (CDA2); 2e = **P27** family pedigree (UH); 2f: **P31** family pedigree (GARDOS); 2 g: **P33** family pedigree (UH); 2 h: **P36** family pedigree (multiple association); 2i: **P40** family pedigree (*ATP11C* hemolysis)

We identified the probable genetic cause of hemolysis in all HS patients **using target genes panel analysis (71 CHA genes) on WES data**. Nineteen patients were heterozygous carriers of one mutation in *ANK1* (8 patients), *SLC4A1* (6 patients), or *SPTB* (5 patients). All patients had different mutations, among which 13 were novel and 8 had already been reported. In one family with typical HS (**P11** and affected relatives), affected members harbored 2 heterozygous probably damaging variations, one in *SPTB* (stop-gained) and the other in *SPTA1* (already-reported missense mutation, previously found in a case of HPP which also harbored the alpha-Lely polymorphism *in trans*). Such an association has rarely been reported in the literature [15, 16, 43, 44]. In 3 families (**P4, P10** and **P11**), genetic tests could be extended to other affected family members and showed cosegregation of mutations and disease (Fig. 2). Two HS patients also had additional mutations in other CHA genes. **P1** carried a heterozygous mutation of alpha-globin gene, called Tunis-Bizerte hemoglobin, responsible for an alpha- thalassemia trait [45]. **P17** carried a variant of uncertain significance (VUS) in *PIEZO1* (p.V860M). In total, 3 out of 20 HS patients (**P1, P11** and **P17**) had variations in two different CHA genes. Patient P2, who had significant iron overload also harbored 2 variants in *HFE* (H63D and C282Y) which had been previously found during iron overload exploration.

### UH GROUP

Twenty patients were classified as **UH** because of discordant or non-contributive clinical features and/or biological tests. Familial segregation study could be performed in 6 families (**P25, P27, P31, P33, P36, P40**, Fig. 2).

Twelve UH patients have been fully characterized (**P21, P22, P25, P29, P30, P31, P32, P36, P37, P38, P39**, and **P40**) by target gene panel analysis of 71 CHA genes on the WES data. One UH patient (**P35**) could be fully characterized thanks to WES analysis. Seven other UH patients have not been fully characterized (**P23, P24, P26, P27, P28, P33** and **P34**) despite extended WES analysis. Interestingly, in those patients WES identified two new possible target genes.

Among the 12 fully characterized UH patients, nine harbored mutations in genes encoding membrane proteins. *SPTA1* was the most frequently mutated gene (in 6 probands: **P21, P22, P29, P36, P37**, and **P39**). Variations in *SPTA1* result in HE, HPP or HS depending on variants type and phase. In **P37** and **P39**, genotypes and phenotypic data (clinical features, blood smear) were suggestive of HPP. We could not clearly decipher between HE or HPP for **P21, 22** and **P29.** Other membrane genes with mutations were *PIEZO1* (**P30** and **P36**, DHSt), *KCNN4* (**P31**, GARDOS MIM 616689), *SPTB* (**P32**, elliptocytosis) and *SLC4A1* (**P36,** SEA ovalocytosis, MIM 166900). Among them, some had original presentation. **P30** had myelodysplasia and important hemolysis without any family history. She carried a constitutional (present in blood and hair bulb DNA) heterozygous *PIEZO1* variant of uncertain significance (VUS) (p.P376A) and had atypical ektacytometry results. **P36** is one of the 3 A/S symptomatic patients explored in our study (**P29, P32** and **P36**). This woman originating from Comoros islands experienced spleen infarct after a long-distance flight and showed the association of a HbS trait, SEA ovalocytosis (*SLC4A1*: p.Ala400_Ala408del), a *G6PD* MATERA A- p.V98M variant at heterozygous state [46], a homozygous *SPTA1* variation (p.E2224D) and a *PIEZO1* mutation (p.R457C) already involved in DHSt [16]. Her daughter (**P36–1**) also presented with hemolytic anemia and harbored the same mutations (the *SPTA1* variant being heterozygous). Her blood smear showed anisopoikilocytosis and some stomatocytes. Another relative (P36–2) was explored and carried the same mutations as **P36–1**. He had a hemolytic anemia and a retinopathy typical of sickle cell disease.

Three fully characterized UH patients had mutations in genes not encoding membrane proteins: *SEC23B* in **P25** with CDA type 2; *CFH* in **P38** with aHUS; *ATP11C* in **P40**. **P25** harbored 2 already described mutations in *SEC23B* gene at compound heterozygous state (Fig. 2) allowing correction of diagnosis towards CDA type 2 and not HS as initially suspected.

**P38** carried a heterozygous mutation in *CFH* (p.R53C), which had previously been found in patients with preeclampsia-related SHUa [47], in complement-related glomerulopathies [48] and in familial forms of AMD (age-related macular degeneration, MIM 610698) [49]. She also carried a heterozygous *PIEZO1* VUS (p.G1416R) but had normal ektacytometry and blood smear. In this case one single mutation in *CFH* probably explains the entire phenotype (AMD, preeclampsia, hemolysis and altered renal function). **P40** carried a likely pathogenic hemizygous variation in *ATP11C* (p.P812S). This X chromosome variation is recorded in gnomAD in only one heterozygous female (no hemizygous males or homozygous females recorded). He also carried one VUS in *ANK1*. His blood smear was normal and ektacytometry showed atypical profile with only dehydration and no change in osmotic resistance. Functional testing of flippase activity is in progress.

Among the 12 fully characterized UH patients, 6 had mutations in several CHA genes:

- **P29***HBB* S mutation and *SPTA1*
- **P31**, *KCNN4 and PIEZO1*
- **P32**, *HBB* S mutation and *SPTB*
- **P36,***HBB* S mutation*, SPTA1, SLC4A1, PIEZO1* and *G6PD*
- **P38,***CFH* and *PIEZO1*
- **P40**, *ATP11C* and *ANK1,*

#### One additional UH patient was characterized thanks to WES analysis

**P35** was found to harbor a heterozygous deleterious variation in *SH2B3*: c.1A > G (initiation codon loss). This result allowed to reconsider diagnosis towards a probable myeloproliferative condition. *SH2B3* somatic mutations have been reported in myeloproliferative neoplasms such as primary myelofibrosis [50]. He also carried a heterozygous VUS in the *SCN9A* gene c.2938G > T (p.A980S) which likely explained the severity of painful crises reported in this patient. *SCN9A* is involved in neurogenic painful syndromes [51].

**Seven UH patients** remained unsolved (**P23, P27** and **P28**) or partially solved (**P24, P26, P33** and **P34**) despite WES extended analysis. **P23** carried a heterozygous *ALAS2* promoter variation (c.-258C > G), which had previously been reported as a cause of X-linked sideroblastic anemia (MIM 300751) [52]. This variation is present in gnomAD at an allelic frequency of 0.54%, with 39 hemizygous males, which suggests that it is most probably a rare benign polymorphism. No other relevant genetic variation was found. **P27** was a female child with major hemolytic anemia at birth and neonatal splenomegaly with thrombocytopenia. Her mother, maternal aunt and maternal grand-father had the same phenotype. She carried a heterozygous variation in *CFH* (p.Q950H) [53], which was absent in the mother and the maternal aunt and is therefore not responsible for CHA in this family. No other relevant genetic variation was found. **P28 was carrier** of two heterozygous variants in two genes involved in CDA: one in *SEC23B* (p.V426I) and one in *CDAN1* (p.P86S). No case of digenic inheritance has yet been reported in CDA. No other relevant genetic variation was found. The other patients had a part of their phenotype explained by WES. In **P24**, candidate gene variations could be identified thanks to WES. A heterozygous missense variation was found in the gene encoding the RNA-specific Adenosine Deaminase (*ADAR)* (p.P529L). No *ADAR* mutation has been associated to date with congenital hemolysis in humans but several studies showed a crucial role for ADAR in mouse erythropoiesis. This patient also carried an apparently homozygous variation of *TRPV4* (p.P638L), present in the gnomAD database at an allelic frequency of 0.03% without any homozygotes recorded (over > 138,000 subjects tested). *TRPV4* mutations have been found in skeletal dysplasia, arthropathies and in a familial form of osteonecrosis [54]. The *TRPV4* mutation could explain osteonecrosis but not hemolysis. **P26** carried *HAMP* and *HFE* variations probably explaining iron overload and *CD46* variation for susceptibility to hemolysis. *HAMP* gene is out of our genes panel analysis. **P33** harbored *G6PD* and *SPTB* variations, which combination cannot explain the severe phenotype. **P34** had UH and iron overload, and variations in 3 genes: a homozygous p.H63D variation in *HFE*, which may contribute to iron overload even though its implication is not totally clear [55, 56], a heterozygous *ABCG8* VUS and 2 heterozygous *ADAMTS13* VUS. *ABCG8* mutations are found in AR sitosterolemia and AD xanthelasma [57]. A single heterozygous *ABCG8* variation is not sufficient to explain hemolysis. This patient had a normal platelet count and ADAMTS13 enzyme activity testing showed a 39% decrease (suggestive of a heterozygous loss-of-function). Normal ADAMTS13 activity in **P34** does not support a diagnosis of AR Thrombotic thrombocytopenic purpura (MIM 274150), and therefore cannot explain hemolysis.

In summary, among n UH patients, 12 patients could be fully characterized thanks to targeted analysis on CHA genes. Eight benefited from extended WES analysis leading to full characterization in one additionnal patient and 7 UH patients not fully characterized but two new potential target genes were identified by WES.

## Discussion

In this study, WES sequencing allowed the identification of a genetic cause of CHA in 33 out of 40 patients (82.5%), including 20 HS patients (100%) and 13 UH patients (65%). All HS patients harbored mutations in already-known HS genes: *ANK1, SLC4A1, SPTB, SPTA1*. All identified variants (21, among which 13 novel) were classified as likely pathogenic or pathogenic loss-of-function mutations. A recent exomic study of 38 Chinese patients with suspected HS found mutations of *ANK1* or *SPTB* in all patients [24]. Gallagher et al in 2019 [25] reported on a group of 24 recessive HS patients explored by WES and whole genome sequencing (WGS), all having mutations found in *SPTA1*. Our results, together with those previously published, suggest that there are probably no other major HS genes. In mouse, beta-adducin deficiency has been shown to cause spherocytosis [58], but no mutations of adducins have yet been identified in human HS.

In UH patients, NGS analysis allowed diagnosis in 13 patients (**P21, P25, P29, P31, P22, P30, P32, P35, P36, P37, P38, P39**, and **P40**). Rate of positivity is lower in UH patients than in HS cases, probably because of the lack of a clear phenotype.

One important finding of our study is the frequent association of several genetic variants in different genes in a same patient, emphasizing the genetic complexity of CHA. Twenty two percents (9/40) of patients harbored several variations in different CHA genes. Among HS patients, only 3 (15%) had variations in other genes, whereas in the UH group, 9/20 (45%) had an association of variants in different CHA genes. The frequent occurrence of multiple genetic variants in single UH patients suggests that this association could contribute to the complexity of the phenotype. Phenotypical tests alone are not able to detect all those variants associations, some of which can have therapeutic consequences (contra-indication of splenectomy in DHSt for example). Such associations of several deleterious variants in genes causing RBC diseases have rarely been reported [59–61] and could be explained in part by the patients’origins. For example, the P36 family that carried variations in 5 CHA genes originated from Comoros Islands, a region of endemic malaria. Plasmodium has been shown to exert a selective pressure on various RBC proteins [62].

Our study also emphasizes the usefulness of exomic approach in the field of CHA, both for discovery of candidate genes (such as *TRPV4* or *ADAR*) and for diagnosis reorientation. Thanks to exomic capture, we reorientated initial diagnosis for **P35** (probable myeloproliferative syndrome) and explained a part of the phenotype in **P24** (osteonecrosis and *TRPV4*), **P26** (*HAMP* and iron overload) and **P35** (painful crisis and *SCN9A*). The cases of **P27** remained unsolved despite large familial investigations. Exomic capture allowed us to find a possible cause for **P24’s** osteonecrosis since this patient harbored a homozygous *TRPV4* variation. *TRPV4* encodes a non-selective calcium-permeant cation channel expressed in different cell types [63] but not in erythrocytes and it has never been associated with hemolysis. Mutations have already been involved in skeletal dysplasia, hereditary neuropathies, arthropathies [64] and recently in a familial case of osteonecrosis [65]. All variants reported were heterozygous and only one paper described a familial case of complex phenotype with severe intellectual disability and neuropathy associated with compound heterozygosity for *TRPV4* mutations [66]. Our patient does not present any neurological symptoms. In this patient, we also found an *ADAR* variation that could be a good candidate for hemolysis. No mutations in *ADAR* have yet been associated with hemolysis in humans. Previous studies in mouse showed that *ADAR* is implicated in erythropoiesis [67, 68]. *ADAR* heterozygous mutations are been found in dyschromatosis symmetrica hereditaria [69] and homozygous or compound heterozygous variations in Aicardi-Goutières syndrome [70].

During the last few years, several NGS studies of CHA have been published [14–24, 26–32, 71] and have shown their usefullness (Additional file 1, Supplementary Table S1). Two recent studies using targeted approaches, focused on 21 index cases and 62 families respectively [16, 17]. They obtained a positive rate (elucidation of the genetic cause of anemia) of 62 and 65% of cases respectively. In our study, this rate was 82.5%. In the two former studies and ours, the positive rate appears to be considerably higher than the WES success rate obtained in other genetic diseases, such as cardiomyopathies or intellectual deficiencies: 62 to 82.5% for CHA versus 30% on average for cardiomyopathies or intellectual deficiencies [72, 73]. The two recent studies of Wang R. et al 2018 and Gallagher et al 2019 reporting WES findings in relatively large CHA samples [24, 25] were only dedicated to HS patient. Our results suggest that WES is highly useful in CHA patients, whether they have HS or UH. Recommendations proposed by King et al in 2015 [13], and compiled by Kim et al in 2017 [74] concerning the indications of genetic testing for CHA diagnosis need to be updated, as they only recommended genetic testing in a limited number of cases and as a second diagnostic step. A recent article of Rets et al in 2019 suggests that targeted NGS becomes the diagnosis standard tool in CHA molecular testing and that WES and WGS could represents the future [75]. Our results show that molecular diagnosis with WES could easily be democratized and be of great help to understand patients’ phenotype, adapting therapeutic approach (such as splenectomy) and to allow genetic counseling. Indeed, exomic approach appeared as particularly useful in UH cases and for complex phenotypes.

## Conclusion

This work emphasizes the usefulness of WES in CHA in order to reach a right diagnosis for each patient. This allows clinical geneticists to provide a personally fitted genetic counseling and specialized clinicians to adjust treatment in some cases. When first-line phenotypic analyses have not been successful in elucidating the disease cause, genetic tests using NGS and especially WES sequencing appears as very helpful to uncover the intricate genetic defects causing CHA and associated manifestations. The recent developments of exomic or genomic sequencing technologies make them the most suitable and cheapest approaches for the genetic diagnosis of these disorders, keeping in mind the absolute necessity of pluridisciplinar teams able to deal with incidental or unexpected findings if needed.

**Fig. 3:**
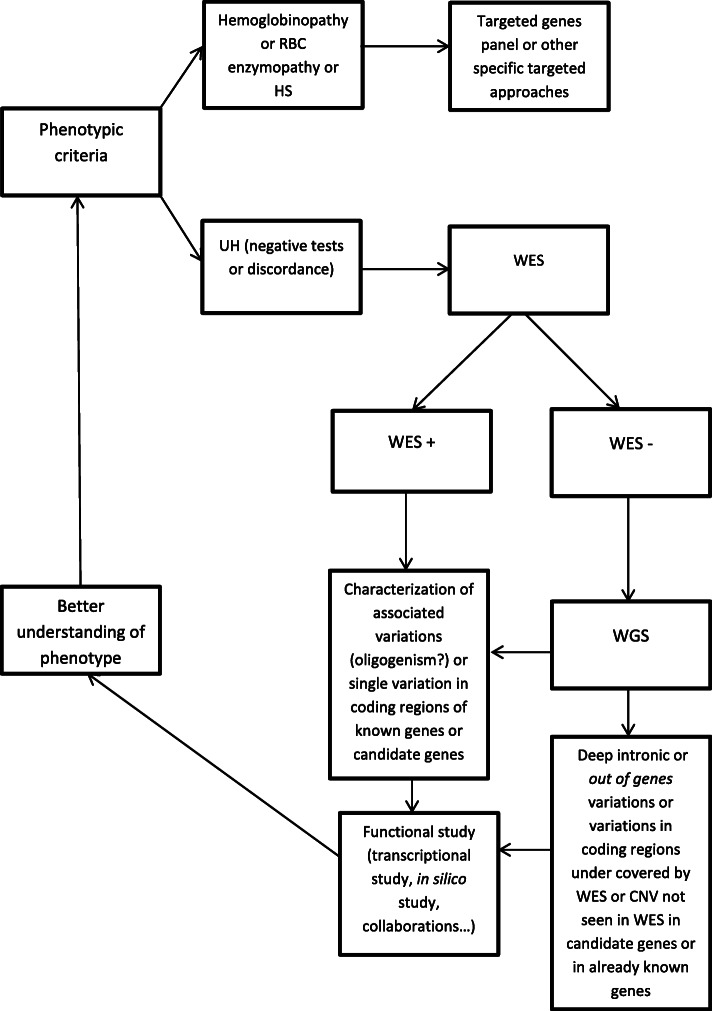
Diagnosis strategy proposition for CHA

Democratization of NGS should lead to update the current recommendations and diagnosis strategy concerning the place of genetic testing in CHA on Fig. 3. The frequent discovery of several mutations in different genes in a same patient also suggests to reconsider the genetic bases of CHA: apparently monogenic diseases may in fact be oligogenic, with satellite variations acting as modulators of the phenotype or resulting in new clinical entities.

Deeper mechanistic studies are warranted to better understand the relationship between genotype and phenotype, especially in patients with atypical or poorly described variants association and to explain VUS. However, some interactions may prove difficult to investigate with currently existing tools. The development of next generation phenotypic tools, such as RBC metabolomics and proteomics, may represent new steps in the exploration of RBC genetic disorders and key tools for variant interpretation.

## Supplementary information

**Additional file 1.**

## Data Availability

The datasets used and/or analysed during the current study are available from the corresponding author on reasonable request.

## Abbreviations

AMD: Age-Related Macular Degeneration
AR: Autosomic recessive
AD: Autosomic dominant
CHA: Congenital hemolytic anemia
DHSt: Dehydrated hereditary stomatocytosis
GFF: Glomerular filtration flow
HS: Hereditary spherocytosis
HPP: Hereditary pyropoïkilocytosis
HE: Hereditary elliptocytosis
MDS: Myelodysplastic syndrome
RBC: Red blood cell
MPE: Membrane protein electrophoresis
MPN: Myeloproliferative neoplasms
NGS: Next generation sequencing
TPI: Triose phosphate isomerase
UH: Unexplained hemolysis
aHUS: Atypical hemolytic and uremic syndrome
VUS: Variant of uncertain significance
WES: Whole exome sequencing
WGS: Whole genome sequencing
XLD: X-linked disorder
