# Supplementary material for: Exome sequencing for diagnosis of congenital hemolytic anemia
Source: Orphanet J Rare Dis. 2020 Jul 8;15:180. doi: 10.1186/s13023-020-01425-5 (PMC7341591; doi:10.1186/s13023-020-01425-5)
Supplement: Supplementary file 1 — Additional file 1. [file 13023_2020_1425_MOESM1_ESM.docx]

Supplementary data

Supplementary table S1 : Recent NGS explorations of CHA, technic, panel, patients

| Reference | Approach **Number of genes in the panel** | Technology | Mean depth of coverage | Number of patients | Positive rate | Comparaison to our panel genes list |
| --- | --- | --- | --- | --- | --- | --- |
| Han J.H. *et al* 2015 | WES | Agilent sureselect exome v4 | 75x | 1 proband | 1/1 |  |
| Lacy J.N. *et al* 2016 | WES | Nimblegen exome enrichment protocol |  | 1 proband with transfuso-dependance non explained | 1/1 |  |
| Roy N.B.A et al 2016 | Targeted panel genes  33 | TSCA (Illumina) | ? | 57 | 38.6% | genes involved in DBA, FA, DKC |
| Agarwal *et al* 2016 | Targeted panel  28 | Haloplex | ? | 15 CHA+ 2 control | 12/17 = 70% | genes involved in congenital hyperbilirubinemia absent in our panel |
| BARRETO R. *et al* 2016 | Targeted panel  40 | Ampliseq | mean coverage 100x | 26 (among them 16 positive control) | 90% of the 10 patients | genes of blood group GYPA and GYPC, PIGT |
| Niss et al 2016 | targeted panel of 12 genes | ? |  | 15 | 15/15 = 100% |  |
| Kim Y. *et al* 2017 | general review |  |  |  |  |  |
| Errichiello et al 2017 | WES | Sureselect exome v5 |  | 2 families, 3 patients with DBA, FA and DKC | 2/2 |  |
| He Y. et al 2017 | 600 genes (list not provided) | Nimblegen | ? | 15 patients from 3 families | 3/3 |  |
| Rongrong Wang et al 2018 | WES | ? | ? | 38 SH cases probable or not clearly diagnosed | 38/38 OK |  |
| Kedar P.S., et al 2018 | Targeted panel  38 gènes | TSCA | ? | 2 | 2/2 with the same homozygous mutation in the GPI gene | double name genes in their list, PGAM2 is not involved in CHA |
| Hamada et al 2018 | WES | ? |  | 10 japanese patients with suspected CDA among them, 7 with targeted sanger sequencing negative --> | 5/7 negative  2/7 with mutations in the G6PD gene and the SPTA1 (HE) |  |
| Khurana et al 2018 | WES | Agilent cinical research exome | 111x | Inherited bone marrow failure syndromes  1 P initially diagnosed as DBA | 2 mutations in the SPTA1 gene --> HPP |  |
| Koker A. et al 2018 | panel |  |  | 1 patient with CDA | 1/1 = 100% |  |
| Ittiwut C et al 2018 | WES | Truseq Agilent |  | 8 thai families with HPP | 7/8 = 87.5% |  |
| Moreno-Carralero MI et al 2018 | Panel | Ion ampliseq Life technologies, Carlsbad, CA, USA) |  | 53 patients with clinical suspicion of CDA (44 families) | 21/53 = 39% | 5 genes of CDA and sanger sequencing of 2 genes |
| Lin P.C. et al 2018 | WES | Truseq exome enrichment kit |  | 7 patients | 4 patients |  |
| Russo R. et al 2018 | Targeted panel  71 gènes ( | Haloplex | ? | 62 families | 64.9% | many genes involved in DBA, CDA (with many candidates genes), auto inflammatory syndromes genes |
| Averbuch N.S. et al 2018 | Targeted panel  76 gènes | SureselectXT target enrichment | pas de données | 21 CHA | 62% | many genes of DBA, CDA, all genes of hemochromatosis |
| Our study | targeted genes analysis involved in CHA | exomic capture Medexome Nimblegen | 150x | 40 :  19 HS  21 unexplained hemolysis | 100 % of HS patients  50% of unexplained hemolysis |  |

Supplementary Table S2 : List of genes involved in AHC and analyzed.

*HK1 (NM_000188), GPI (NM_000175), PFKM (NM_000289, NM_001166687, NM_001166688), ALDOA (NM_000034, NM_184041, NM_001243177, NM_001127617, NM_184043), TPI1 (NM_000365), PGK1 (NM_000291), ENO1 (NM_00142), PKLR (NM_181871, NM_000298), PGAM1 (NM_002629), BPGM (NM_199186, NM_001293085, NM_001724), SLC2A1 (NM_006516), GALT (NM_000155), G6PD (NM_000402.4), PGD (NM_001304451, NM_001304452, NM_002631), CYB5R3 (NM_000398, NM_001171661, NM_001129819 , NM_007326, NM_001171660), GSR (NM_000637), GSS (NM_000178), GPX1 (NM_000581), GCLC (NM_001498), NT5C3A (NM_001002010.2), AK1 (NM_000476.2), GATA1 (NM_002049.3), HBB (NM_000518.4), HBA1 (NM_000558.4), HBA2 (NM_000517.4), HBG1 (NM_000559.2), HBG2 (NM_000184.2), CDAN1 (NM_138477.2), C15ORF41 (NM_001290233.1), SEC23B (NM_001172745.1), KLF1 (NM_006563.3), ANK1 (NM_020476.2), SPTB (NM_001024858.3), SPTA1 (NM_003126.2), SLC4A1 (NM_000342.3), EPB42 (NM_000119.2), EPB41 (NM_001166005.1), PIEZO1 (NM_001142864.3), KCNN4 (NM_002250.2), EPB72 (STOM) (NM_004099.5), RHAG (NM_000324.2), RHCE (NM_020485.4), ABCG5 (NM_022436.2), ABCG8 (NM_022437.2), LCAT (NM_000229.1), XK (NM_021083.2), ADAMTS13 (NM_139025.4), HF1 (NM_000186.3), CFHR3 (NM_021023.5), CFHR1 (NM_002113.2), CD46 (MCP) (NM_172359.2), CFI (NM_000204.3), C3 (NM_000064.3), THBD (NM_000361.2), DGKE (NM_003647.2), CD59 (NM_203330.2), FOXP3 (NM_014009.3), ITK (NM_005546.3), TNFRSF6 (FAS) (NM_000043.4), FASLG (NM_000639.2), CASP10 (NM_032977.3), LRBA (NM_006726.4), STIM1 (NM_001277961.1), UROS (NM_000375.2), CPOX (NM_000097.5), HMOX1 (NM_002133.2), ATP11C (NM_173694)*

| Gene Symbol | Numéro HGNC |
| --- | --- |
| HK1 | 4922 |
| GPI | 4458 |
| PFKM | 8877 |
| ALDOA | 414 |
| TPI1 | 12009 |
| PGK1 | 8896 |
| ENO1 | 3350 |
| PKLR | 9020 |
| PGAM1 | 8888 |
| BPGM | 1093 |
| SLC2A1 | 11005 |
| GALT | 4135 |
| G6PD | 4057 |
| PGD | 8891 |
| CYB5R3 | 2873 |
| GSR | 4623 |
| GSS | 4624 |
| GPX1 | 4553 |
| GCLC | 4311 |
| NT5C3A | 17820 |
| AK1 | 361 |
| ADA | 186 |
| GATA1 | 4170 |
| HBB | 4827 |
| HBA1 | 4823 |
| HBA2 | 4824 |
| HBG1 | 4831 |
| HBG2 | 4832 |
| CDAN1 | 1713 |
| C15ORF41 | 26929 |
| SEC23B | 10702 |
| CDAN3 | 1715 |
| KLF1 | 6345 |
| ANK1 | 492 |
| SPTB | 11274 |
| SPTA1 | 11272 |
| SLC4A1 | 11027 |
| EPB42 | 3381 |
| EPB41 | 3377 |
| PIEZO1 | 28993 |
| KCNN4 | 6293 |
| STOM | 3383 |
| RHAG | 10006 |
| RHCE | 10008 |
| SLC4A1 | 11027 |
| ABCG5 | 13886 |
| ABCG8 | 13887 |
| LCAT | 6522 |
| XK | 12811 |
| ADAMTS13 | 1366 |
| CFH | 4883 |
| CFHR3 | 16980 |
| CFHR1 | 4888 |
| CD46 | 6953 |
| CFI | 5394 |
| CFB | 1037 |
| C3 | 1318 |
| THBD | 11784 |
| DGKE | 2852 |
| CD59 | 1689 |
| FOXP3 | 6106 |
| ITK | 6171 |
| FAS | 11920 |
| FASLG | 11936 |
| CASP10 | 1500 |
| LRBA | 1742 |
| STIM1 | 11386 |
| UROS | 12592 |
| CPOX | 2321 |
| HMOX1 | 5013 |
| ATP11C | 13554 |

**Supplementary table S3 :** Variants per gene and *in silico* analysis

| Patient | *Gene* | HGVS Coding | HGVS Protein | Variant type | status | MAF gnomAD | Reference | Functional protein domain prediction with SMART Software | Polyphen-2 | Sift | Mutation Taster | Align GVGD | MaxEntScan  HSF |
| --- | --- | --- | --- | --- | --- | --- | --- | --- | --- | --- | --- | --- | --- |
| **P1** | ***ANK1 NM_020476.2*** | c.5152C>T | p.Gln1718* | STOP-GAINED | het | Absent | Not described | probable mRNA decay | NA | NA | NA | NA | NA |
| **P2** | ***ANK1 NM_020476.2*** | c.1702-2A>C |  | splicing | het | Absent | Not described | NA | NA | NA | NA | NA | MES : -100%  HSF : -100%  Abolition of acceptor site Exon 17 skipping probable |
| **P6** | ***ANK1 NM_020476.2*** | c.5497C>T | p.Arg1833* | STOP-GAINED | het | Absent | Hayette *et al* 1998 Ankyrine St Etienne 2 | probable mRNA decay | NA | NA | NA | NA | NA |
| **P21** | ***ANK1 NM_020476.2*** | c.1801-17G>A |  | SPLICING | het | Absent | Duru *et al* 1992 et Edelman *et al* 2007 | NA | NA | NA | NA | NA | Probable creation of cryptic acceptor splicing site score HSF = 80,2% |
| **P9** | ***ANK1 NM_020476.2*** | c.4462C>T | p.R1488* | STOP-GAINED | het | Absent | Ozcan *et al* 2003 | probable mRNA decay | NA | NA | NA | NA | NA |
| **P10**  **P10-1** | ***ANK1 NM_020476.2*** | c.1A>G | p.? | ATG suppression | het | Absent | Not described | NA | NA | NA | NA | NA | NA |
| **P13** | ***ANK1 NM_020476.2*** | c.534delC | p.H178Qfs*75 | frameshift | het | Absent | Not described | probable mRNA decay | NA | NA | NA | NA | NA |
| **P16** | ***ANK1 NM_020476.2*** | c.712-2G>A |  | splicing | het | Absent | Not described | NA | NA | NA | NA | NA | Probable exon 8 skipping and truncated protein production |
| **P40** | ***ANK1 NM_020476.2*** | c.4558G>C | p.Glu1520Gln | missense | het | 0.0021% | Not described | Unknown function | benign | tolerated | disease causing | C0 | No predicted splicing effect |
| **P3** | ***SLC4A1 NM_000342.2*** | c.1458C>G | p.Y486* | STOP-GAINED | het | Absent | Not described | probable mRNA decay | NA | NA | NA | NA | NA |
| **P4**  **P4-1** | ***SLC4A1 NM_000342.2*** | c.486-2A>G |  | SPLICING | het | Absent | Not described | NA | NA | NA | NA | NA | MES : -100%  HSF : -100%  Probable exon 7 skipping STOP premature in exon 8 |
| **P7** | ***SLC4A1 NM_000342.2*** | c.1322T>G | p.L441R | Missense (no splicing effect) | het | Absent | Not described | HCO3- cotransporter domain | probably damaging | deleterious | disease causing | : Align GVGD : C45 | No predicted splicing effect |
| **P15** | ***SLC4A1 NM_000342.2*** | c.1462G>A | p.V488M | missense | het | Absent | Alloisio *et al* 1997 | HCO3- cotransporter domain | probably damaging | tolerated | disease causing | C0 |  |
| **P36**  **P36-1** | ***SLC4A1 NM_000342.2*** | c.1199_1225del | (p.A400_A408del) | SEA Ovalocytosis | het | gnomAD all : 0.0047% | Wilder *et al* 2009 | HCO3- cotransporter domain | NA | NA | NA | NA | NA |
| P17 | ***SLC4A1 NM_000342.2*** | c.2423G>A | (p.R808H) | missense | het | Absent | Bogardus *et al* 2012 | HCO3- cotransporter domain | Probably damaging | Deleterious | Disease causing | C25 | No predicted splicing effect |
| P18 | ***SLC4A1 NM_000342.2*** | c.2279G>A | (p.R760Q) | missense | het | Absent | Jarolim *et al* 1995 | HCO3- cotransporter domain | Probably damaging | Deleterious | Disease causing | C35 | No predicted splicing effect |
| P5 | *SPTB*  *NM_001024858* | c.1331_1338del | p.L444Pfs*3 | frameshift | het | Absent | Dhermy *et al* 1998 | probable mRNA decay | NA | NA | NA | NA | NA |
| P11  P11-1  P11-2 | *SPTB*  *NM_001024858* | c.2863C>T | p.R955* | STOP-gained | het | Absent | Not described | probable mRNA decay | NA | NA | NA | NA | NA |
| P12 | *SPTB*  *NM_001024858* | c.4973+5G>A |  | splicing | het | Absent | Not described | NA | NA | NA | NA | NA | MaxEnt: -100.0% HSF: -14.6% exon 23 skipping? |
| P32 | *SPTB*  *NM_001024858* | c.6706C>A | p.L2236M | Missense | het | Absent | Not described | Pleckstrin homology domain. | Benign | Deleterious | Disease causing | C0 | No predicted splicing effect |
| P32 | *SPTB*  *NM_001024858* | c.6737C>T | p.A2246V | Missense | het | Absent | Not described | Pleckstrin homology domain. | Probably damaging | Tolerated | Polymorphism | C0 | No predicted splicing effect |
| P20 | *SPTB*  *NM_001024858* | c.3916C>T | p.R1306* | STOP-gained | het | 1/246265 | Not described | probable mRNA decay | NA | NA | NA | NA | NA |
| P14 | *SPTB*  *NM_001024858* | c.5623C>T | p.Gln1875* | STOP-gained | het | Absent | Not described | probable mRNA decay | NA | NA | NA | NA | NA |
| P33  P33-1 | *SPTB*  *NM_001024858* | c.6271C>A | p.P2091T | Missense Beginning exon (splicing?) | het | gnomAD All : 0,0065% | Not described | Unknown region | Benign | Tolerated | Disease causing | C0 | No predicted splicing effect, but 2^nd^ base of exon, ARN needed |
| P19 | *SPTB*  *NM_001024858* | c.3436dup | p.L1146Pfs*36 | frameshift | het | absent | Not described | probable mRNA decay | NA | NA | NA | NA | NA |
| P19 | *SPTB*  *NM_001024858* | c.6101G>A | p.S2034N | missense | het | 0.00041% | Not described | spectrin repeat domain | benign | deleterious | disease causing | C45 | No predicted splicing effect, |
| P21 | *SPTA1 NM_003126.3* | c.6600+5G>T |  | splicing | het | Absent | Not described | NA | NA | NA | NA | NA | MES : -62,6%  HSF : -13.6%  Probable Abolition of donor splicing site of intron 47, RNA study needed |
| P29 | *SPTA1 NM_003126.3* | c.1688G>A | p.R563Q | missense with a probable predicted effect on splicing | het | 0.11% | Not described | spectrin repeat forms a three-helix bundle | Probably damaging | Tolerated | Disease causing | C0 | Possible creation of cryptic acceptor splicing site score HSF 83% vs 85%WT effect possible on protein : truncated of 4 AA |
| P11  P11-1  P11-2 | *SPTA1 NM_003126.3* | c.6421C>T | p.R2141W | Missense | het | 0.20% | Niss *et al* 2016 | spectrin repeat forms a three-helix bundle | Probably damaging | Deleterious | Disease causing | C0 |  |
| P22 | *SPTA1 NM_003126.3* | c.2898G>A | p.= | SPLICING | het | Absent | Not described | NA | NA | NA | NA | NA | MaxEnt: -29.3% HSF:-10.8% last base of exon 20  ARN study in progress |
| P36  P36-1 | *SPTA1 NM_003126.3* | c.6672A>C | (p.E2224D) | Missense | hom  het | 1.5% gnomAD homoz = 0 | Not described | spectrin repeat forms a three-helix bundle | Probably damaging | Deleterious | Disease causing | C35 | No predicted splicing effect |
| P37 | *SPTA1 NM_003126.3* | c.3291G>A | (p.W1097*) | STOP-GAINED | het | Absent | Not described | probable mRNA decay | NA | NA | NA | NA | NA |
| P39 | *SPTA1 NM_003126.3* | c.779T>C | p.Leu260Pro | missense | het | 0.0014% (Afr 0.017%) | Marchesi S.L *et al* 1987 | effect on spectrin tetramer formation | probably damaging | deleterious | disease causing | C0 | No predicted splicing effect |
| P23 | *ALAS2*  *NM_000032.4* | c.-258C>G het |  | promotor | het | gnomAD All : 0,54% | Bekri *et al* 2003 | NA | NA | NA | NA | NA | NA |
| P25 | *SEC23B*  *NM_001172745.2* | c.40C>T | (p.R14W) | missense | het | gnomAD All : 0,022% | Russo *et al* 2011 | Unknown region | Possibly damaging | Deleterious | Disease causing | C0 | No predicted splicing effect |
| P28 | *SEC23B*  *NM_001172745.2* | c.1276G>A | (p.V426I) | missense | het | 4.33% | Schwartz *et al* 2009 | Sec23/Sec24 beta-sandwich domain | Benign | Tolerated | Polymorphism | C0 | No predicted splicing effect |
| P25 | *SEC23B*  *NM_001172745.2* | c.325G>A | (p.E109K) | missense | het | gnomAD All : 0,023% | Russo *et al* 2011 | Unknown region | Probably damaging | deleterious | Disease causing | C0 | No predicted splicing effect |
| P28 | *CDAN1*  ***NM_138477*** | c.256C>T | p.P86S | missense | het | 0.052% | Not described | Internal repeat 1 | Benign | Tolerated | Polymorphism | C0 | No predicted splicing effect |
| P26 | *HAMP NM_021175.2* | c.49_54del | p.L17_L18del | deletion | het | Absent | Not described | prepropeptide | NA | NA | NA | NA | No predicted splicing effect |
| P26 | *CD46 NM_172359* | c.402T>G | (p.I134M) | missense | het | Absent | Not described | Unknown region | Possibly damaging | Deleterious | Polymorphism | C0 | No predicted splicing effect |
| P27 | *CFH NM_00186.3* | c.2850G>T | p.Q950H | missense | het | gnomAD All : 0,39% | Mohlin *et al* en 2015 | complement control protein domain | Benign | Deleterious | Polymorphism | C0 | No predicted splicing effect |
| P38 | *CFH NM_00186.3* | c.157C>T | p.R53C | missense | het | 0.0014% | Fakhouri *et al* 2010  Servais *et a*l 2012 | complement control protein domain | probably damaging | deleterious | disease causing | C0 | No predicted splicing effect |
| **P30** | ***PIEZO1***  *NM_001142864.2 :* | c.1126C>G | p.P376A | missense | het | Absent | Not described | Unknown region | Benign | Tolerated | Polymorphism | C0 | Creation of possible cryptic acceptor site score HSF 82,7 vs 86,07 WT probable premature stop codon in exon 10  ARN study in progress |
| **P31** | ***PIEZO1***  *NM_001142864.2* | c.3629C>T | p.A1210V | missense | het | gnomAD All : 0,006% | Not described | Unknown region | Benign | Tolerated | Disease causing | C0 | No predicted splicing effect |
| **P36**  **P36-1** | ***PIEZO1***  *NM_001142864.2* | ***c.1369C>T*** | ***p.R457C*** | missense | het | Absent | Russo *et al* 2018 | transmembrane helix region | Probably damaging | Deleterious | Disease causing | C0 | No predicted splicing effect |
| **P17** | ***PIEZO1***  *NM_001142864.2* | ***c.2578G>A*** | ***p.V860M*** | missense | het | gnomAD : 0.0028% | Not described | Unknown region | Possibly damaging | Tolerated | Disease causing | C0 | No predicted splicing effect |
| **P38** | ***PIEZO1***  *NM_001142864.2* | ***c.4246G>A*** | ***p.G1416R*** | missense | het | gnomAD : 0.0033% | Not described | extracellular domain linking 2 Transmembrane domains | Probably damaging | Deleterious | benign | C0 | Possible creation of a cryptic acceptor site |
| P31  P31-1 | *KCNN4 NM_002250.2* | c.1055G>A | p.R352H | missense | het | Absent | Rappetti Mauss *et al 2015* | Calmodulin binding domain | Possibly damaging | Deleterious | Disease causing | C0 |  |
| P33  P33-1 | *G6PD NM_ 000402.4 :* | c.538G>A | p.V180I | missense | het | Absent | Not described | NAD-binding domain of glucose-6-phosphate dehydrogenase | possibly damaging | Deleterious | disease causing | C25 | No predicted splicing effect |
| P36 | *G6PD NM_ 000402.4 :* | c.292G>A | p.V98M | missense | het | gnomAd All : 1.15% | Vulliamy *et al* 1988 |  | Probably damaging | tolerated | NA | C0 | No predicted splicing effect |
| **P34** | ***ABCG8*** *NM_022437 :* | c.-27G>A |  | 5’UTR | het | Absent | Not described | NA | NA | NA | NA | NA | NA |
| **P34** | ***ADAMTS13*** *NM_139025 :* | c.4007G>A | (p.R1336Q) | missense | het | gnomAD all : 0,0012%, | Not described | CUB domain (for complement C1r/C1s, Uegf, Bmp1) | Benign | Deleterious | Disease causing | C35 | No predicted splicing effect |
| **P34** | ***ADAMTS13*** *NM_139025 :* | c.119C>G | (p.Ala40Gly) | missense | het | gnomAD 0,00041% | Not described | Unknown region | Benign | Tolerated | polymorphism | C0 | creation possible donor splicing site HSF 78.4 vs 77 canonic site |
| P26 | *HFE* NM_000410.3 | c.845G>A | (p.C282Y) | missense | het | All : 3,37% ; | Many references | Immunoglobulin C-Type 1 domain | Probably damaging | Deleterious | Polymorphism | C65 | No predicted splicing effect |
| **P34**  **P37** | *HFE* NM_000410.3 | c.187C>G | (H63D) | missense | hom | All : 10.83% | Many references | MHC_I domain | benign | tolerated | Polymorphism | C0 | No predicted splicing effect |
| **P35** | ***SH2B3***  ***NM_005475.2*** | ***c.1A>G*** | ***p.?*** | ATG loss | het | Absent | Not described | ATG initiator | NA | NA | NA | NA | NA |
| **P24** | ***ADAR NM_001111.4*** | c.1586C>T | p.P529L | missense | het | Absent | Not described | Double-Stranded RNA-binding Motif domain | Probably damaging | deleterious | Disease causing | C65 |  |
| **P24** | ***TRPV4***  ***NM_021625.4*** | c.1913C>T | (p.P638L) | missense | hom | gnomAD = 0.03%, no  homozygote recorded | Not described | Ion transport domain | Benign | Tolerated | Disease causing | C0 | No predicted splicing effect |
| **P1** | ***HBA1 NM_0005558.3*** | **c.389T>C** | **p.L130P** |  | **het** | absent | Darbellay R *et al* 1995 | domain globin | probably damaging | Deleterious | Disease causing | C0 | No predicted splicing effect |
| **P35** | ***SCN9A***  ***NM_002977.3*** | c.2938G>T | (p.A980S**)** | missense | het | absent | Not described | Sodium ion transport-associated domain | Possibly damaging | Deleterious | Disease causing | C0 | No predicted splicing effect |
| **P40** | ***ATP11C NM_173694.4*** | c.2434C>T | (p.Pro812Ser) | missense | hem | 0.0011%  (1/57660)  no hemizygous nor homozygous | Not described | P-type ATPase, transmembrane domain superfamily | probably damaging | tolerated | Disease causing | C0 | No predicted splicing effect |

Supplementary table S3 legend: List of variants per gene with HGVS coding name and protein name, variant type, status (het: heterozygous state; hom: homozygous state; hem: hemizygous state), minor allele frequency in gnomAD database (<https://gnomad.broadinstitute.org/>); reference if it exists, functional protein domain predicted by SMART (<http://smart.embl-heidelberg.de/>), *in silico* prediction for missense variants using Polyphen-2 (<http://genetics.bwh.harvard.edu/pph2/>), SIFT (<https://sift.bii.a-star.edu.sg/>), Mutation taster (<http://www.mutationtaster.org/>), and Align GVGD ([http://agvgd.iarc.fr/, scores classified from C0 to C65 from benign to deleterious) and](http://agvgd.iarc.fr/,%20scores%20classified%20from%20C0%20to%20C65%20from%20benign%20to%20deleterious)%20and) for splicing variant MaxEnt Scan (<http://genes.mit.edu/burgelab/maxent/Xmaxentscan_scoreseq.html>) and Human splice finder (<http://www.umd.be/HSF/>); ; NA: not applicable

Supplementary table S4 : clinvar accession numbers of new variants

Supplementary Table S5 : Clinical and biological description of patients with RBC membrane study results

| **Patient number, gender, geographic origin**  **Initial diagnosis** | **Clinical and**  **Family history** | **Membrane study**  EMA decrease of fluorescence  Ektacytometry  Protein membrane electrophoresis |
| --- | --- | --- |
| **P1, M, Europe/Maghreb**  **Hereditary spherocytosis** | Splenomegaly, neonatal hyperbilirubinemia,  No family history of hemolysis | -35%  HS  Abnormal ankyrin synthesis |
| **P2, M, France**  **Hereditary spherocytosis** | Stroke in 2014, family history of hereditary spherocytosis with dominant transmission and iron overload with phlebotomy | -29%  HS  ND |
| **P3, M, Portugal**  **Hereditary spherocytosis** | **Splenomegaly,** important newborn jaundice, gallstone, ironoverload, PNH negative  No family history of hemolysis | -30%  HS  Decrease of protein Band 3 |
| **P4, F, Asia**  **(affected sister P4-1)**  **Hereditary spherocytosis** | Iron overload**.**  One sister with the same phenotype | -31%  ND  ND |
| **P5, M, France**  **Hereditary spherocytosis** | Cholecystectomy and splenectomy,  Family history of hereditary spherocytosis | -34%  -ND  ND |
| **P6, F, Europe**  **Hereditary spherocytosis** | **Hepatosplenomegaly**, transfusionnal exchange at birth,  Family history of hereditary spherocytosis with dominant transmission | -26%  ND  ND |
| **P7, M, Europe**  **Membrane disorder** | Multiple renal cysts, Lapeyronie disease, iron overload needing phlebotomy  Family history : 1 brother with the same phenotype | -28%  HS  ND |
| **P8, M, Europe**  **Hereditary spherocytosis** | Splenomegaly, cholecystectomy , iron overload needing **phlebotomy**  Family history : 1 sister with the same phenotype | -18%  ND  ND |
| **P9, M, Europe/Italy**  **Hereditary spherocytosis** | Splenectomy, iron overload needing **phlebotomy**  Familial case | -24%  HS  ND |
| **P10, M, Europe**  **(P10-1 affected brother)**  **Atypical hereditary spherocytosis with massive iron overload** | Important iron overload needing **phlebotomy**, family history, father and one brother with the same phenotype | -24%  HS  ND |
| **P11, F, Europe**  **Affected son P11-1**  **Affected daughter P11-2**  **Hereditary spherocytosis** | Splenectomy in proband and son  Familial Hereditary Spherocytosis | -27%  HS  Decrease of spectrin alpha and beta, decrease of  protein  4.2 and ankyrin |
| **P12, M, Europe**  **newborn with Hereditary spherocytosis** | Neonatal icterus in a context of familial h**ereditary spherocytosis with dominant transmission** | ND  ND  ND |
| **P13, F, Maghreb**  **Sporadic Hereditary spherocytosis** | **Sporadic hereditary spherocytosis**, chronical anemia since childhood, treated by speciafoldine, splenomegaly, gallstones🡪cholecystectomia, recent transfusion  No family history | -26%  HS  Protein 4.2 decrease but recent transfusion |
| **P14, F, Europe**  **Sporadic Hereditary spherocytosis** | Sporadic case, no family history of hemolysis | -36%  HS  ND |
| **P15, F, Italy/Europe**  **Hereditary spherocytosis** | One grand mother with the same phenotype | -32%  ND  ND |
| **P16, F, Maghreb**  **Hereditary spherocytosis** | Hereditary spherocytosis in one affected daughter | -27%  HS  ND |
| **P17, F, Asia (Laos)**  **Hereditary spherocytosis** | Family history of hemolysis | -18%  HS  ND |
| **P18, F, Europe**  **Hereditary spherocytosis** | Family history unknown | - 39%  HS  ND |
| **P19, F, Maghreb**  **hereditary spherocytosis** | Familial hereditary spherocytosis, splenectomized, thrombocytosis, 2 asymptomatic daughters | -22%  HS  ND |
| **P20, M, Europe**  **Sporadic Hereditary spherocytosis?** | Familial HS, revealed during primoinfection with EBV needing 2 CGR transfused, splenomegaly (22cm), father transfused at birth  1 paternal uncle with neonatal icterus | -30%  HS  normal |
| **P21, F, Europe**  **Hemolysis on Membrane disorder** | Splenectomy, no hemolysis since splenectomy  Family history : 1 son with the same phenotype | -10%  ND  ND |
| **P22, F, Europe**  **Atypical Hereditary spherocytosis** | **Important** abdominal pain +++  Splenectomized, pain persists after splenectomy, PNH negative  No family history of hemolysis | normal  HS  Normal |
| **P23, F, Europe**  **(P23-1 healthy daughter) Congenital dyserythropoiesis** | History of miscarriages, Iron overload and dyserythropoiesis with anemia(6g/dl) on ParvovirusB19 infection during pregnancy, 1 transfusion, ironoverload confirmed on hepatic MRI needing **phlebotomy**  No family history of hemolysis | normal  normal  ND |
| **P24, M, Europe**  **Huge hemolysis with osteonecrosis** | Multiple osteonecrosis without sickle cell anemia  1 son with arthropathy, 1 maternal uncle with multiple orthopedic surgeries | normal  ND  ND |
| **P25, M, Maghreb**  **P25-1 Father**  **P25-2 Mother**  **Unexplained hemolysis** | Abdominal pain since 2014, cholecystectomy, hepatic steatosis, splenomegaly, microlithiasis,  Cholecystectomy on his mother, no consanguinity  No family history of hemolysis | -9%  HS  atypical profile of decrease of band 3 |
| **P26, M, Europe**  **Xerocytosis and iron overload** | **Phlebotomy due to iron overload**  No family history of hemolysis | ND  ND  ND |
| **P27, F, Europe**  **P27-1 : mother**  **P27-2 : maternal aunt**  **Neonatal unexplained hemolysis** | Important hemolytic anemia at birth, important neonatal splenomegaly, thrombocytopenia, polytransfusions during first life year,  Mother, maternal aunt and maternal grand father with the same phenotype | Normal  Normal  ND |
| **P28**, **M, Maghreb**  **Unexplained hemolysis** | No family history of hemolysis | ND  ND  ND |
| **P29, M, Europe**  **Hemolysis +++ on A/S patient** | Sickle cell trait with hemolysis +++, splenomegaly +++, oesophageal varices, no iron overload, No family history of hemolysis | -7%  Atypical with isolated osmotic resistance  ND |
| **P30, F, Africa**  **Myelodysplasia**  **Unexplained hemolysis** | Myelodysplasia was showed on bone marrow evaluation with multilineage abnormalities and atypical large erythroblastic hyperplasia (72%), myeloid caryotype normal, somatic myeloid NGS found 1 pathogenic variant in *EZH2* at 12.5%  Important Hemolysis  **No family history** | increasing of fluorescence  Atypical : increasing in osmotic resistance without red  blood cell dehydration  ND |
| **P31, M, Italy**  **GARDOS**  **P31-1, M, affected son** | Hemolysis on 3 generations, every people with hemolysis has been splenectomized, important iron overload | Normal  Atypical, sub-normal  ND  Normal  Atypical, sub-normal  ND |
| **P32, F, Africa**  **A/S + hemolysis** | A/S with hemolysis and slight anemia, No family history | Normal  Atypical  ND |
| **P33, F, Europe**  **P33-1, mother**  **P33-2, unaffected father** | Neonatal icterus until D7, at 2 months important hemolytic anemia with high reticulocytes count needing several transfusions, blood smear normal,  Well compensated hemolysis  No hemolysis | Normal  Normal  ND  ND  Normal  ND  ND  ND  ND |
| **P34, M, Europe**  **Unexplained hemolysis** | Iron overload needing **phlebotomy**  Family history unknown | -5%  ND  ND |
| **P35, M, Europe**  **Unexplained hemolysis** | Unexplained sporadic hemolysis , important painful splenomegaly, neurogenic pain of arms and legs, advanced COPD, No family history of hemolysis | -5%  Sub- normal  ND |
| **P36, F, Comoros islands**  **A/S with Unexplained hemolysis**  **P36-1 : affected daughter** | Abdominal painful crisis after a long-haul flight, sickle cell trait with hemolytic anemia, with icterus  One daughter with hemolytic anemia | -23%  ND  ND  -23%  flat profile : SEA ovalocytosis  ND |
| **P37, M, Europe**  **TPI deficiency?** | Chronic hemolytic anemia since childhood with exacerbation during infectious diseases, No family history and no history of neonatal icterus | EMA Normal but after recent transfusion  In favor of erythrocytic membranopathy (context of recent transfusion)  ND |
| **P38, F, Europe**  **unexplained hemolysis** | Iron overload with asthenia Hypothyroïdy, age related macular degeneration, 2 pregnancies with late pre eclampsia and normal birth weight babies, Moderate jaundice since childhood during infectious diseases, ,alteration of renal function, chronic hemolysis  Mother with the same phenotype : DMLA and hemolytic crisis | -5%  normal  ND |
| **P39, F, Africa**  **unexplained hemolysis** | Suspected elliptocytosis or HPP, one daughter with important hyperbilirubinemia at birth needing transfusion | -7%  Elliptocytosis with probable alpha thal trait  ND |
| P40, M, Maghreb  unexplained hemolysis | Sporadic case, splenomegaly, multiple endocrine troubles, insipidus diabete, well-compensated hemolysis | -5%  atypical HS  ND |

Table S5 Legend: This table summarizes clinical and biological data available for all 40 patients. Results of membrane red blood cell exploration are detailed when available. M: male, F: female; COPD (Chronic Obstructive Pulmonary Disease), ND: not done; EMA: eosin-5-maleimide; HS: hereditary spherocytosis; DHSt: dehydrated hereditary stomatocytosis; GFF: glomerular filtration flow; HPP: hereditary pyropoïkilocytosis

**Supplementary table S6 :** Biological results of all patients

| Patient | Relatives | Sex | Haptoglobin g/L | LDH IU/L | P50 mmHg | RBC 106/µL | Hb (g/dl) | Ht (%) | MCV (fL) | MCH pg | MCHC (g/dL) | RDW (%) | PLT (G/L) | Reticulocyte  G/L | Total bilirubin  µM | Unbound bilirubin µM | EMA test | Density red cells | Ferritin  µg/L | G6PD  activity  (UI/g Hb) | PK  Activity  (UI/g Hb) | HbA2 % | HbF % | HbA1c % |
| --- | --- | --- | --- | --- | --- | --- | --- | --- | --- | --- | --- | --- | --- | --- | --- | --- | --- | --- | --- | --- | --- | --- | --- | --- |
| P1 |  | M | <0.1 | 169 | 19.6 | 5.4 | 14.1 | 40 | 75 | 26 | 35 | 18 | 238 | 431 | 26 | 9 | -35% | 1.102 | 560 | 22.3 | 14.9 | 2.5 | 0.3 | 3.1 |
| P2 |  | M | <0.1 | 199 | 26 | 5 | 16.1 | 45 | 89 | 32 | 36 | 14 | 242 | 148 | 42 |  | -29% | 1.104 | 775 | 17 | 19 | 3 | 2.2 | 3.6 |
| P3 |  | M | <0.1 | 175 | 26.8 | 4.6 | 15.6 | 44 | 95 | 34 | 36 | 16 | 147 | 246 | 30 | 21 | -30% | 1.106 | 843 | 15.7 | 16.3 | 2.7 | 2.1 | ND |
| P4 |  | F |  | 221 | 28.2 | 3.5 | 11.8 | 33 | 94 | 34 | 36 | 16 | 170 | 183 | 16 | 11 | -31% | 1.109 | 192 | 10.6 | 20.1 | 2.3 | 1.9 | 4 |
|  | P4-1 : affected sister | F | <0.1 | 225 | 26.4 | 4.4 | 13.6 | 38 | 86 | 31 | 36 | 16 | 197 | 223 | 11 | 6 | -32% | 1.100  10% of dense cells | 343 | 8.8 | 14.8 | 2.9 | 0.7 | 4.3 |
| P5 |  | M | <0.1 | 196 | 28.1 | 4.5 | ND | 42 | 92 | ND | ND | 18 | 279 | 310 | 76 | 68 | -34% | 1.100 |  | 16.8 | 24.5 | 3.2 | 1.1 | ND |
| P6 |  | F | <0.1 | 177 | 28.6 | 3.6 | 11.9 | 33 | 91 | 33 | 36 | 19 | 210 | 300 | 12 | 8 | -26% | 1.098 | 136 | 17 | 27.8 | 2.7 | 1.4 | 3.1 |
| P7 |  | M | <0.1 | 291 | 27.6 | 4.6 | 15.7 | 44 | 95 | 34 | 36 | 14 | 163 | 196 | 15 | 10 | -28% | 1.106 | 128 | 12.5 | 12.3 | ND | ND | ND |
| P8 |  | M | <0.1 | 315 | 27.7 | 3.8 | 12.3 | 34 | 89 | 32 | 36 | 17 | 123 | 239 | 30 | 20 | ND | 1.103 | 46* | 18.4 | 25.5 | 2 | 0.7 |  |
| P9 |  | M | 0.9 | * | 27.8 | 5.9 | 16.8 | 48 | 81 | 29 | 36 | 15 | 413 | 178 | 11 | 7 | -24% | 1.108 | 302 | 19.1 | 25.4 | 2.7 | 1.6 | 3.1 |
| P10 |  | M | <0.1 | 299 | 28.4 | 3.3 | 13 | 37 | 110 | 39 | 35 | 18 | 200 | 394 | 37 | 24 | -24% | 1.103 | 1740 | ND | ND | 2.9 | 2.2 |  |
|  | P10-1 : affected brother | M | 1.6 (with CRP >5 mg/l) | 148 | ND | 5.1 | 15.5 | 43 | 85 | 30 | 36 | 14 | 447 | 140 | 7 | 3 | -32% | 1.102  8% of dense cells | 676 | 14.5 | 28.2 | 2.8 | 0.5 | ND |
| P11 |  | F | 1.2 | 247 | 27.5 | 4.9 | 15.9 | 43 | 88 | 32 | 37 | 14 | 340 | 218 | 11 | 6 | -27% | 1.103 | ND | 11.8 | 13.9 | 2.3 | 0.5 | ND |
|  | P11-1 : affected son  splenectomized | M | 0.1 | 176 | 25.4 | 5.5 | 16.9 | 46 | 83 | 30 | 37 | 13 | 418 | 188 | 20 | 18 | -33% | 1.103  30% of dense cells | 228 | 12.5 | 17.5 | 2.7 | 0.5 | ND |
|  | P11-2 affected daughter | F | <0.1 | 216 | 24.3 | 3.2 | ND because of hemolysis | 27 | 84 |  |  | 21 | 274 | 477 | 96 | 85 | -38% | 1.108 35% of dense cells | 143 | 14.9 | 27.4 | 2.7 | 0.5 | 2.5 |
| P12 |  | M | ND | ND | ND | 5.19 | 18.5 | 50.6 | 97.5 | 36.6 | 35.6 | 18.5 | 315 | ND | 188.1 | 179.5 | ND | ND | ND | ND | ND | ND | ND | ND |
| P13 |  | F | <0.1 | 318 | 26.2 | 3.9 | 11.7 | 33 | 87 | 30 | 36 | 18 | 400 | 633 | 39 | 27 | -26% | 1.108 | 292 | 17.2 | 30.8 | 2.9 | 0.5 |  |
| P14 |  | F | ND | ND | ND | 3.94 | 10.1 | 29.6 | 75 | 25 | 34 | ND | 349 | 709 | ND | ND | -36% | ND | ND | ND | ND | 2.7 | 1.6 | ND |
| P15 |  | F | <0.1 | 197 | 28.1 | 4.3 | 13.3 | 37 | 86 | 31 | 36 | 16 | 252 | 198 | 45 | 34 | -32% | 8% of dense cells | ND | ND | ND | ND | ND | ND |
| P16 |  | F | <0.1 |  | 28.1 | 3.2 | 10.4 | 29 | 91 | 33 | 36 | 18 | 286 | 477 | 20 | 13 | -27% | 1.105  33% of dense cells | 720 | 17.3 | 26.6 | 2.6 | 2.4 | 2.9 |
| P17 |  | F | <0.1 |  | 26.8 | 2.1 | 7.5 | 21 | 102 | 36 | 36 | 28 | 79 | 455 | 29 | 19 | ND | ND | 58 | 21.8 | 35.6 | 2.7 | 0.5 | 2.7 |
| P18 |  | F | <0.1 | 221 | 27.4 | 4.4 | 13.8 | 36 | 83 | 32 | 38 | 18 | 167 | 302 | 43 | 30 | -39% | 1.105, 27% of denses cells | 98 | 13.5 | 20.6 | 2.8 | 0.5 | 4 |
| P19 |  | F | 1.6 | 159 | ND | 4.9 | 12 | 37 | 75 | 24 | 32 | 18 | 919 | 132 | 5 | 3 | -22% | nd | 18 | 14.8 | 38.1 | 2.2 | 0.5 | 5.3 |
| P20 |  | M | ND | 200 | 27.7 | 4.5 | 12.7 | 35 | 79 | 28 | 36 | 19 | 267 | 328 | 28 | 19 | -30% | 1.093 | 275 | 18.8 | 31.1 | 3 | 0.5 | ND |
| P21 |  | F | 0.7 | 190 | 26.8 | 4.8 | 14.5 | 43 | 91 | 31 | 34 | 14 | 268 | ND | 194 | 185 | -10% | 1.101 | 61 | 17.7 | 15.8 | 2.8 | 1.4 | ND |
| P22 |  | F | <0.1 | 169 | ND | 3.8 | 12.5 | 35 | 91 | 33 | 36 | 16 | 167 | 204 | 22 | 14 | normal | ND | ND | 14.4 | 20.2 | 2.7 | 1.1 | ND |
| P23 |  | F | 1.2 | 129 |  | 4.4 | 14 | 39 | 87 | 32 | 36 | 13 | 260 | 67 | 12 |  | Normal | 1.105 19% of dense cells | ND | 10.1 | 14.9 | 2.7 | 0.5 | 6.1 |
|  | P23-1 healthy daughter | F | 1.1 | ND | ND | 4.5 | 13.1 | 38 | 83 | 29 | 35 | 12 | 152 | 47 | ND | ND | ND | 1.097 | 68 | 14 | 17.5 | 3 | 1.2 | ND |
| P24 |  | M | <0.1 | 232 | 28 | 5.3 | 15.5 | 47 | 88 | 29 | 33 | 13 | 268 | 45 | 8 | 5 | normal | 1.093 | 165 | 18.1 | 19 | 3.2 | 0.5 | ND |
| P25 |  | M | <0.1 | 175 | 25.9 | 4.5 | 14.7 | 41 | 91 | 32 | 36 | 15 | 141 | 243 | 55 | 42 | -9% | 1.105 | 368 | 15 | 19.4 | 2.9 | 0.5 | ND |
|  | P25-1 : father | M | ND | ND | ND | 5.2 | 15 | 44 | 85 | 29 | 34 | 14 | 192 | 67 | ND | ND | ND | 1.098 | ND | 14.2 | 18.6 | 2.7 | 0.5 | 6.6 |
|  | P25-2 : mother | F | 2.2 | ND | ND | 4.5 | 14.1 | 41 | 90 | 31 | 35 | 12 | 207 | 54 | ND | ND | ND | 1.098 | 46 | 13.2 | 17 | 2.9 | 0.8 | ND |
| P26 |  | M | 0.1 | 126 | 27 | 5.4 | 17.1 | 48 | 88 | 31 | 36 | 14 | 166 | 79 | 16 | 11 | ND | 1.105 | 202 | 13 | 12.8 | 2.9 | 1.1 | ND |
| P27 |  | F | <0.1 | ND | ND | ND | ND | ND | ND | ND | ND | 57 | ND | ND | ND | ND | ND | ND | ND | 16.8 | 24.8 | ND | ND | ND |
|  | P27-1 : mother | F | ND | ND | ND | ND | ND | ND | ND | ND | ND | ND | ND | ND | ND | ND | ND | ND | ND | ND | ND | ND | ND | ND |
|  | P27-2 : maternal aunt | F | ND | ND | ND | ND | ND | ND | ND | ND | ND | ND | ND | ND | ND | ND | ND | ND | ND | ND | ND | ND | ND | ND |
| P28 |  | M | <0.1 | 169 | ND | 4.3 | 13.8 | 39 | 91 | 32 | 35 | 14 | 157 | 34 | 3 | 1 | ND | 1.098 | 252 | 10.3 | 10.5 | 2.3 | 3.9 | ND |
| P29  A/S |  | M | <0.1 | 312 | 27.7 | 6.5 | 15.7 | 48 | 74 | 24 | 32 | 20 | 375 | 65 | 73 | 63 | -7% | 1.092 | 139 | 10.7 | 24.7 | 3.3 | 0.4 | ND |
| P30 |  | F | <0.1 | 152 | ND | 2.6 | 8.5 | 26 | 100 | 33 | 33 | 20 | 281 | 86 | 10 | 6 | normal | 1.088 | 471 | 30.3 | 13.8 | 2 | 7.6 | ND |
| P31 |  | M | <0.1 | 319 | 27.9 | 3.3 | 11.9 | 34 | 104 | 36 | 35 | 15 | 464 | 711 | 86 | 74 | normal | 1.098 | 829 | ND | ND | 1.9 | 0.7 | 3.9 |
|  | P31-1 affected son | M | <0.1 | 203 | 29.2 | 2.4 | 9.6 | 27 | 112 | 40 | 36 | 16 | 662 | 353 | 161 | 143 | normal | 1.095  With 9% of dense cells | 1369 | 18.9 | 30.9 | 2.1 | 2.4 | 3.9 |
| P32  A/S |  | F | <0.1 | 244 | 33.6 | 3.5 | 11.9 | 34 | 98 | 34 | 35 | 13 | 169 | 57 | 9 | 5 | -11% | 1.099 | 281 | 12.8 | 13.3 | 3.3 | 1.9 | ND |
| P33 |  | F | ND | ND | ND | ND | 4.7 | ND | 104 | ND | ND | ND | ND | 325 | 39 | 30 | ND | ND | ND | 22.8 | 63 | ND | ND | ND |
|  | P33-1 : affected mother | F | <0.1 | ND | ND | ND | ND | ND | ND | ND | ND | ND | ND | ND | ND | ND | ND | ND | ND | 10.9 | 23.7 | ND | ND | ND |
|  | P33-2 : healthy father | M | normal | ND | ND | ND | ND | ND | ND | ND | ND | ND | ND | ND | ND | ND | ND | ND | ND | 11.8 | 13 | ND | ND | ND |
| →P34 |  | M | <0.1 | 231 | 27.5 | 5 | 16 | 45 | 89 | 32 | 36 | 14 | 184 | 190 | 7 | 5 | -5% | 1.100 10% of dense cells | 37 | 12.1 | 13.1 | 2.7 | 0.5 | 5 |
| P35 |  | M | <0.1 | ND | 26.8 | 5.1 | 15.9 | 46 | 90 | 31 | 34 | 14 | 259 | 343 | 23 | 15 | -5% | 1.096 5% of dense cells | 180 | 16.6 | 24.2 | 2.3 | 0.5 | 3.9 |
| P36 |  | F | <0.1 | ND | ND | 3 | 9.8 | 28 | 92 | 32 | 35 | 19 | 203 | 73 | 78 | 71 | -23% | 1.1032  A/S  Abnormal shape of the curve | 34 | 16.6 | 12.5 | 3.3 | 2.4 | ND |
|  | P36-1 | F | <0.1 | 166 | ND | 3.7 | 10.5 | 31 | 85 | 28 | 34 | 24 | 318 | 119 | 39 | 9 | -23% | ND | 16 | 16.3 | 17.7 | 3.2 | 0.7 | ND |
| P37 |  | M | <0.1 | ND | 28.3 | 2.1* | 7.3 | 21 | 102 | 35 | 35 | 26 | 83 | 227 | 134 | 122 | Normal * | 1.094 abnormal shape of the curve in favor of membrane disorder | 365 | 21.8 | 21.7 | 2.6 | 1.1 | ND |
| P38 |  | F | <0.1 | 245 | 28.7 | 3.9 | 12.9 | 37 | 96 | 33 | 34 | 12 | 206 | 112 | 9 | 3 | normal (-5%) | 1.100  abnormal profile shifted on the right | 463 | 12.3 | 22.8 | 2.6 | 0.5 | 3.9 |
| P39 |  | F | <0.1 | 216 | ND | 5.2 | 10 | 32 | 62 | 19 | 31 | 40 | 256 | 76 | 26 | 8 | -7% | 1.090  curve shifted on the left | 565 | 17.3 | 25.6 | 2.6 | 0.5 | 4.1 |
| P40 |  | M | <0.1 | 130 | ND | 4.6 | 14.7 | 42 | 90 | 32 | 35 | 13 | 173 | 107 | 35 | 25 | -5% | ND | 74 | ND | ND | ND | ND | ND |

**Supplementary table S6** Legend: Biological results of all patients. M: male; F: female; ND = not done. Normal values are available in Supplementary Table

**Supplementary Table S7 : Normal biological values**

|  | **Normal values** |
| --- | --- |
| **Total Bilirubin (µM)** | <21 |
| **Free Bilirubin (µM)** | <17.6 |
| **Conjugated Bilirubin (µM)** | <3.4 |
| **Haptoglobin g/L** | 0.3-2.0 |
| **P50 mmHg** | 24-28 |
| **LDH (IU/l)** | <250 |
| **Ferritin (µg/l)** | 30-400 |
| **P50 (mmHg)** | 24-28 |
| **Leucocytes (G/L)** | 4-10 |
| **Red blood cell count (T/L)** | 4.6-6.2 |
| **Hb (g/dl)** | 13-17 |
| **Hematocrit %** | 39-51 |
| **MCV (fl)** | 80-100 |
| **MCH (pg)** | >27 |
| **MCHC %** | 32-36 |
| **RDW** | <15 |
| **Reticulocytes (G/L)** | 20-120 |
| **Thrombocytes (G/L)** | 150-400 |
| **G6PD activity (UI/g Hb)** | 11-17 |
| **PK activity (UI/g Hb)** | 14-19 |
| **HbA2** | 2.1-3.1 % |
| **HbF** | <1 % |
| **HbA1c** | 4-6% |
| **EMA test** | fluorescence decrease <16% : absence of membrane red blood cell abnormality  fluorescence decrease >21% : presence of a probable membrane red blood cell abnormality |
| **density of red blood cells** | 1.0905<N<1.0975 |
| **percentage of dense red blood cells** | <1% |

**µM = micromoles per liter; IU/l = international unit per liter ; µg/l = microgram per liter ; g/l = gram per liter ; nM = nanomoles per liter ; G/l = giga per liter; T/l = tera per liter ; fl = flemtoliter ; pg = pictogram ; g/dl = gram per deciliter ; mM = milimoles per liter**

Table S8: HS patients and relatives blood smear evaluation and genetic result

| Patient | Peripheral blood smear | Genetic analysis results |
| --- | --- | --- |
| P1 | Many spherocytes, anisocytosis, microcytosis, rare giant platelets | *ANK1 NM_020476.2:* c.5152C>T  (p.Gln1718*) het exon 39  *HBA1* NM_0005558.3*:* c.389T>C, (p.L130P) het exon 3  (Hb Tunis-Bizerte) |
| P2 | Some spherocytes, anisopoïkilocytosis | *ANK1* NM_020476.2*:* c.1702-2A>C het intron 15  *HFE* NM_000410.3  c.187C>G (p.H63D) het exon 2  c.845G>A (p.C282Y) het exon 4 |
| P3 | Some spherocytes, anisopoïkilocytosis | *SLC4A1* NM_000342.2*:* c.1458C>G, (p.Y486*) het exon 13 |
| P4 | Some spherocytes, anisocytosis | *SLC4A1* NM_000342.2: c.486-2A>G het Intron 6 |
| P4-1 affected sister | Anisochromia, rare spherocytes | *SLC4A1* NM_000342.2: c.486-2A>G het Intron 6 |
| P5 | Many spherocytes, anisopoïkilocytosis | *SPTB*NM_001024858*:* c.1331_1338del, (p.Leu444Profs*3) het exon 10 |
| P6 | Many spherocytes | *ANK1* NM_020476.2*:* c.5497C>T  (p.R1833*) het exon 41 |
| P7 | Many spherocytes | *SLC4A1* NM_000342.2: c.1322T>G, (p.L441R) het exon 12 |
| P8 | Many spherocytes, anisopoïkilocytosis | *ANK1* NM_020476.2*:* c.1801-17G>A het  Intron 16 |
| P9 | Some Jolly bodies, anisopoikilocytosis, spherocytes | *ANK1* NM_020476.2*:* c.4462C>T (p.R1488*) het exon 37 |
| P10 | Many spherocytes, Jolly bodies, anisopoikilocytosis | *ANK1* NM_020476.2*:* c.1A>G (p.?) het exon 1 |
| P10-1 affected brother | anisopoïkilocytosis, Jolly bodies, spherocytes, acanthocytes, rare giant platelets | *ANK1* NM_020476.2*:* c.1A>G (p.?) het exon 1 |
| P11 | Poikilocytosis, Jolly bodies, spherocytes, acanthocytes, pappenheimer bodies, heinz body juxta membrane on blood smear | *SPTB* NM_001024858: c.2863C>T (p.R955*) het exon 15  *SPTA1* NM_003126.3: c.6421C>T (p.R2141W) het exon 45 |
| P11-1  Affected son | Poikilocytosis, Jolly bodies, spherocytes, acanthocytes pappenheimer bodies, heinz body juxta membrane on blood smear | *SPTB* NM_001024858: c.2863C>T (p.R955*) het exon 15  *SPTA1* NM_003126.3: c.6421C>T (p.R2141W) het exon 45  + Alpha-lely het *trans* |
| P11-2  Affected daughter | many spherocytes, some acanthocytes, anisocytosis | *SPTB* NM_001024858: c.2863C>T (p.R955*) het exon 15  *SPTA1* NM_003126.3: c.6421C>T (p.R2141W) het exon 45  + Alpha-lely het *trans* |
| P12 | ND | *SPTB* NM_001024858: c.4973+5G>A het intron 23 |
| P13 | Many spherocytes, anisocytosis | *ANK1* NM_020476.2: c.534delC (p.H178Qfs*75)  Het exon 6 |
| P14 | Spherocytes and pappenheimer bodies | *SPTB* NM_001024858.2: c.5623C>T (p.Q1875*) het exon 26 |
| P15 | Anisocytosis, polychromasy, many spherocytes | *SLC4A1* NM_000342.2: c.1462G>A (p.V488M) het exon 13 |
| P16 | Many spherocytes, anisocytosis | *ANK1 NM_020476.2:*  c.712-2A>G het intron 7 |
| P17 | ND | *SLC4A1* NM_000342.2*:* c.2423G>A (p.R808H) het exon 18  *PIEZO1 :* c.2578G>A (p.V860M) het exon 19 |
| P18 | anisopoikilocytosis, spherocytes, bite red cells | *SLC4A1* NM_000342.2*:* c.2279G>A (p.R760Q) het exon 17 |
| P19 | anisopoikilocytosis, microcytosis, hypochromia, jolly bodies, spherocytes, acanthocytes | *SPTB* NM_001024858.2*:* c.3436dup (p.L1146Pfs*36) het exon 15  and  c.6101G>A (p.S2034N) het exon 29 |
| P20 | Many spherocytes, anisopoikilocytosis | *SPTB* NM_001024858.2: c.3916C>T (p.R1306*)  Het exon 18 |

Legend table S8 : het = heterozygous, hom = homozygous; ND = not done; HS = hereditary spherocytosis

Supplementary Table S9: UH patients and relatives blood smear evaluation and genetic result

| Patient | Peripheral blood smear | Genetic analysis results |
| --- | --- | --- |
| P21 | Poikilocytosis, acanthocytes | *SPTA1* NM_003126.3: c.6600+5G>T het Intron 47 /  c.6531-12C>T (alpha lely) het intron 45 |
| P22 | Bite red cells, anisopoikilocytosis, no spherocytes | *SPTA1* NM_003126.3: c.2898G>A p.(=) het exon 20  *SPTA1* NM_003126.3 Alpha Lely het |
| P23 | normal | *ALAS2* NM_000032.4*:*  c.-258C>G het promoter |
| P23-1  Healthy daughter | normal | Absence of *ALAS2* variant |
| P24 | Poikilocytosis  Rare target cells | *Negative on CHA genes panel analysis*  *TRPV4* NM_021625*:* c.1913C>T (p.P638L) hom exon 13  *ADAR* NM_001111*:* c.1586C>T (p.P529L) het exon 2 |
| P25 | Rare schistocytes, anisopoikilocytosis, polychromasia, anisochromia | *SEC23B* NM_001172745.2: c.40C>T (p.R14W) het Exon 2/  c.325G>A (p.E109K) het Exon 4 |
| P25-1  father | normal | *SEC23B* NM_001172745.2: c.325G>A (p.E109K) het Exon 4 |
| P25-2  mother | normal | *SEC23B* NM_001172745.2 : c.40C>T (p.R14W) het Exon 2 |
| P26 | anisocytosis | *HAMP* NM_021175.2  c.49_54del (p.L17_L18del) het Exon 1  *HFE* NM_000410.3:  c.845G>A (p.C282Y) het Exon 4  *CD46* NM_172359:  c.402T>G (p.I134M) het Exon 4 |
| P27 | some spherocytes | *CFH NM_00186.3:*  c.2850G>T  (p.Q950H) het Exon 18 |
| P27-1  Affected mother | ND | Absence of *CFH* variant |
| P27-2  Affected maternal aunt | ND | Absence of *CFH* variant |
| P28 | ND | *SEC23B* NM_001172745.2: c.1276G>A (p.V426I) het exon 11  *CDAN1* NM_138477 : c.256C>T (p.P86S) het exon 2 |
| P29  A/S | Anisopoikilocytosis, microcytosis, hypochromia, some schistocytes | *SPTA1* NM_003126.3: c.1688G>A (p.R563Q) het exon 14  and c.6531-12C>T het Alpha LELY intron 45 |
| P30 | Anisopoikilocytosis, target cells, macrocytosis, polychromasia, schistocytes | *PIEZO1* NM_001142864.2: c.1126C>G (p.P376A) het Exon 10 |
| P31 | Anisopoikilocytosis, giant platelet, Jolly bodies, anisochromia, acanthocytes, basophile granulations, target cells, macrocytosis | *KCNN4* NM_002250.2: c.1055G>A (p.R352H) het exon 7  *PIEZO1* NM_001142864.2: c.3629C>T (p.A1210V) het exon 25 |
| P31-1  Affected son | Anisocytosis, poikylocytose, polychromasie, macrocytosis, target cells, jolly bodies, spherocytes, hemoglobin repartition ablormality | *KCNN4* NM_002250.2: c.1055G>A (p.R352H) het exon 7  Absence of *PIEZO1* variant |
| P32  A/S | Very rare sickle cells, target cells, polychromasia | *SPTB* NM_001024858.2 : c.[6706C>A ; 6737C>T]  p.[L2236M ; A2246V]  exon 33 |
| P33 | No schistocytes, no spherocytes | *G6PD* NM_ 000402.4:  c.538G>A (p.V180I) het exon 5  *SPTB* NM_001024858.2 : c.6271C>A (p.P2091T) het exon 31 |
| P33-1  mother | ND | *G6PD* NM_ 000402.4:  c.538G>A (p.V180I) het exon 5  *SPTB* NM_001024858.2 : c.6271C>A (p.P2091T) het exon 31 |
| P33-2  Unaffected father | ND | Absence of *G6PD* and *SPTB* variants |
| →P34 | anisocytosis | *HFE* NM_000410.3: c.187C>G (p.H63D) hom exon 2  *ABCG8* NM_022437: 5’UTR c.-27G>A het  *ADAMTS13* NM_139025: c.119C>G (p.Ala40Gly) het exon 2/ c.4007G>A (p.R1336Q) het exon 28 |
| P35 | Stomatocytes, poïkilocytosis | Negative on genes panel analysis  *SCN9A* NM_002977.3: c.2938G>T (p.A980S) het exon 17  *SH2B3* NM_005475.2: c.1A>G (p. 0?) het exon 2 |
| P36 | Anisopoïkilocytosis, hemighosts, abnormal hemoglobin repartition | *SPTA1*NM_003126.3*:* c.6672A>C (p.E2224D) hom exon 48  *SLC4A1* NM_000342.2*:* c.1199_1225del (p.A400_A408del) het exon 11  *PIEZO1*NM_001142864.2 *:* c.1369C>T (p.R457C) het exon 12  *G6PD* NM_000402 : c.292G>A (p.V98M) het exon 4  and c.466A>G (p.N156D) het exon 5 |
| P36-1  Affected daughter | anisopoikilocytosis, some stomatocytes | *SPTA1*NM_003126.3*:* c.6672A>C (p.E2224D) het exon 48  *SLC4A1* NM_000342.2*:* c.1199_1225del (p.A400_A408del) het exon 11  *PIEZO1*NM_001142864.2 *:* c.1369C>T (p.R457C) het exon 12  Absence of *G6PD* variant |
| P37 | erythrocytes fragments, dacryocytes, basophiles punctuations, polychromatophily | *SPTA1*NM_003126.3*:* c.3291G>A (p.W1097*) het exon 23  c.6531-12C>T alpha lely het  *HFE*NM_000410.3: c.187C>G (p.H63D) hom exon 2 |
| P38 | normal | *CFH NM_00186.3*: c.157C>T (p.R53C) het exon 2  *PIEZO1*NM_001142864.2: c.4246G>A (p.G1416R) het exon 31 |
| P39 | anisopoikilocytosis, microcytosis, hypochromia, many elliptocytes, many erythrocytes fragments | *SPTA1*NM_003126.3 *:* c.779T>C (p.L260P) het exon 6  c.6531-12C>T het intron 45 |
| P40 | normal | *ATP11C* NM_173694*:* c.2434C>T (p.P812S) hem exon 21  *ANK1* NM_020476*: c.4558G>C (p.E1520Q) het exon 38* |

Table S9 legend: hem = hemizygous, het = heterozygous, hom= homozygous; ND = not done; UH = unexplained hemolysis

Supplementary Table S10: Molecular study results and variants description in hereditary spherocytosis patients group

| Patient, sex, origin  Initial diagnosis 🡪diagnostic post NGS | **Variation**  Gene NM  Nomenclature  c. (p.)  Status  exon | Allelic Frequency in open source database  gnomAD | *In silico* study of missense variations  Polyphen 2  Mutation taster  Sift  Align GVGD | Splicing in silico study  MaxEntScan (MES)  HSF (Human splice finder) | Family study  Functionnal study  Literature reference  Local study | variant Class according to ACMG guidelines | Membrane study  EMA  Ektacytometry  Protein membrane electrophoresis |
| --- | --- | --- | --- | --- | --- | --- | --- |
| **P1, M, Europe/Maghreb**  **Hereditary spherocytosis---> HS** | ***ANK1 NM_020476.2:* c.5152C>T**  **(p.Gln1718*) het exon 39**  ***HBA1 NM_0005558.3:* c.389T>C, (p.L130P) het exon 3**  **(Hb Tunis-Bizerte)** | Absent  Absent | Probable mRNA decay |  | Darbellay R *et al* 1995 | pathogenic  likely pathogenic | -35%  compatible with HS  Abnormal ankyrin synthesis |
| **P2, M, France**  **Hereditary spherocytosis--> HS** | ***ANK1 NM_020476.2:* c.1702-2A>C het intron 15**  ***HFE* NM_000410.3**  c.187C>G (p.H63D) het exon 2  c.845G>A (p.C282Y) het exon 4 | Absent  gnomAD : 10.83%  gnomAD All : 3,37% | benign  tolerated  Polymorphism  C0  Probably damaging  Deleterious  Polymorphism  C65 | MES : -100%  HSF : -100%  Abolition of acceptor site probable exon 17 skipping  No splicing effect predicted  No splicing effect predicted | RNA study : use of cryptic acceptor splicing site in exon 16 --> probable deletion of 19 AA in ANK1 protein  Kaczorowska-Hac *et al* 2016 | pathogenic  uncertain significance  pathogenic | -29%  compatible with HS  ND |
| **P3, M, Europe**  **Hereditary spherocytosis or stomatocytosis --> HS** | ***SLC4A1 NM_000342.2:* c.1458C>G, (p.Y486*) het exon 13** | Absent | Probable mRNA decay |  |  | pathogenic | -30%  HS  Decrease protein Band 3 |
| **P4, F, Asia**  **(sister P4-1)**  **Hereditary spherocytosis--> HS** | ***SLC4A1* NM_000342.2: c.486-2A>G het Intron 6** | Absent |  | Probable exon 7 skipping with premature STOP codon in exon 8 | Sister with the same phenotype carries the same heterozygous variant  RNA study : absence of mutated transcript : confirmation of mRNA decay | pathogenic | -31%  ND  ND |
| **P5, M, France**  **Hereditary spherocytosis--> HS** | ***SPTB*NM**_**001024858*:* c.1331_1338del, (p.Leu444Profs*3) het exon 10** | Absent | Probable mRNA decay |  | Dhermy *et al* 1998  Mutated transcript is not expressed : mRNAdecay | pathogenic | -34%  ND  ND |
| **P6, F, Europe**  **Hereditary spherocytosis--> HS** | ***ANK1 NM_020476.2:* c.5497C>T**  **(p.R1833*) het** exon 41 | Absent |  |  | Hayette *et al*  1998: Ankyrine St Etienne 2  No mRNA decay, production of truncated protein | pathogenic | -26%  ND  ND |
| **P7, M, Europe**  **Membrane disorder, DHSt?** 🡪HS | ***SLC4A1* NM_000342.2**: c.1322T>G, (p.L441R) het exon 12 | Absent | probably damaging  disease causing  deleterious  C45 | No splicing effect predicted | Located into the 2^nd^ TMD of band 3 protein | likely pathogenic | -28%  HS  Significative decrease of band 3 |
| **P8, M, Europe**  **Hereditary spherocytosis--> HS** | ***ANK1 NM_020476.2:*** c.1801-17G>A het  Intron 16 | Absent |  | Creation of a cryptic acceptor splicing site predicted by HSF | Described by Duru *et al* and Edelman *et al*  Creation of a cryptic acceptor splicing site | likely pathogenic | -18%  ND  ND |
| **P9**, **M, Europe/Italy**  **Hereditary spherocytosis--> HS** | ***ANK1 NM_020476.2:*** c.4462C>T (p.R1488*) het exon 37 | Absent | Probable mRNA decay |  | Ozcan *et al* 2003, description associated with hereditary spherocytosis | pathogenic | - 24%  HS  ND |
| **P10, M, Europe**  **(P10-1 = affected brother)**  **Atypical hereditary spherocytosis with massive iron overload --> HS** | ***ANK1 NM_020476.2:*** c.1A>G (p.?) het exon 1 | Absent | Loss of ATG traduction initiator codon |  | Brother with the same phenotype carrying the *ANK1* variant in heterozygous state confirmed in Sanger | pathogenic | -24%  HS  ND |
| **P11, F, Europe**  **Affected son P11-1**  **Affected daughter P11-2**  **Hereditary spherocytosis --> HS** | ***SPTB*** **NM**_**001024858**: c.2863C>T (p.R955*) het exon 15  ***SPTA1*** **NM_003126.3**: c.6421C>T (p.R2141W) het exon 45 | Absent  gnomAD All : 0,20% | Probable mRNA decay  Probably damaging  Disease causing  Deleterious  C0 |  | Family study : son and daughter have the same genotype with also alpha Lely polymorphism in compound heterozygous state  Described by Niss *et al* 2016 | pathogenic  likely pathogenic | -27%  HS  ND |
| **P12, M, Europe**  **Hereditary spherocytosis --> HS** | ***SPTB*** **NM**_**001024858**: c.4973+5G>A het intron 23 | Absent |  | MaxEnt: -100.0% HSF: -14.6%  Probable deleterious effect on splicing |  | likely pathogenic | ND  ND  ND |
| **P13, F, Maghreb**  **Hereditary spherocytosis --> HS** | ***ANK1***  ***NM_020476.2***: c.534delC (p.H178Qfs*75)  het  exon 6 | Absent | Probable mRNA decay |  |  | pathogenic | -26%  HS  protein 4.2 decrease |
| **P14, F, Europe**  **Hereditary spherocytosis--> HS** | ***SPTB*** **NM_001024858.2**: c.5623C>T (p.Q1875*) het exon 26 | Absent | Probable mRNA decay |  |  | pathogenic | -36%  HS  ND |
| **P15, F, Italy/Europe**  **Hereditary spherocytosis --> HS** | ***SLC4A1* NM_000342.2**: c.1462G>A (p.V488M) het exon 13 | gnomAD All : 0.00041% |  |  | Described by Alloisio *et al* 1997 | pathogenic | -32%  ND  ND |
| **P16, F, Maghreb**  **Hereditary spherocytosis --> HS** | ***ANK1 NM_020476.2:***  c.712-2A>G het intron 7 | Absent |  | MaxEnt: -100.0%  HSF: -100.0% | Abolition of canonical acceptor splicing site of intron 7, an exon 8 skipping is highly probable leading to the production of a truncated protein | pathogenic | -27%  HS  ND |
| **P17, F, Asia (Laos)**  **Hereditary spherocytosis --> HS** | ***SLC4A1* NM_000342.2*:*** c.2423G>A (p.R808H) het exon 18  ***PIEZO1 :*** c.2578G>A (p.V860M) het exon 19 | Absent  gnomAD : 0.0028% | Probably damaging  Disease causing  Deleterious  C25  Possibly damaging  Disease causing  Tolerated  C0 | No predicted effect on splicing  No predicted effect on splicing | Bogardus *et al* 2012 | likely pathogenic  uncertain significance | -18%  HS  ND |
| **P18, F, Europe**  **Hereditary spherocytosis --> HS** | ***SLC4A1* NM_000342.2*:*** c.2279G>A (p.R760Q) het exon 17 | absent | Probably damaging  Disease causing  Deleterious  C35 | No predicted effect on splicing | Jarolim *et al* 1995 : absence of mutated protein at the red cell membrane surface | Pathogenic | -39%  HS  ND |
| **P19, F, Maghreb**  **hereditary spherocytosis -->HS** | ***SPTB*** **NM_001024858.2*:*** c.3436dup (p.L1146Pfs*36) het exon 15  and  c.6101G>A (p.S2034N) het exon 29 | absent  gnomAD : 0.00041% | frameshift  benign  damaging  disease causing  C45 | No predicted effect on splicing  No predicted effect on splicing | probable mRNA decay | Likely pathogenic  uncertain significance | -22%  HS  ND |
| **P20, M, Europe**  **Hereditary spherocytosis --> HS** | ***SPTB***  **NM_001024858.2**: c.3916C>T (p.R1306*)  het  exon 18 | Absent | Probable mRNA decay |  |  | likely pathogenic | -30%  HS  normal |

Table S10 Legend: Variants description and classification according to ACMG guidelines. *In silico* study of missense variations was assessed thanks to Polyphen-2, Mutation taster, Sift and Align GVGD. HGMD professional and pubmed web interface were used to check for variants description. Abbreviations: het: heterozygous state; hom: homozygous state; F: female; M: male; HS: hereditary spherocytosis; gnomAD: genome agregation database <https://gnomad.broadinstitute.org> ; ND: not done

Supplementary Table S11: Molecular study results and variants description of unexplained hemolysis patients.

| Patient, sex, origin  Initial diagnosis 🡪diagnostic post NGS | **Variation**  Gene NM  Nomenclature  c. (p.)  Status  exon | Allelic Frequency in open source database  gnomAD | *In silico* study of missense variations  Polyphen 2  Mutation taster  Sift  Align GVGD | Splicing in silico study  MaxEntScan (MES)  HSF (Human splice finder) | Family study  Functionnal study  Literature reference  Local study | variant Class according to ACMG guidelines | Membrane study  EMA  Ektacytometry  Protein membrane electrophoresis |
| --- | --- | --- | --- | --- | --- | --- | --- |
| **P21, F, Europe**  **Hemolysis on Membrane disorder** 🡪HE or HPP | ***SPTA1*** **NM_003126.3**: c.6600+5G>T het Intron 47 /  c.6531-12C>T(alpha lely) het intron 45 | Absent/  25% |  | MES : -62,6%  HSF : -13.6%  Probable loss of donor splicing site of intron 47 |  | likely pathogenic | -10%  ND  ND |
| **P22, F, Europe**  **Atypical Hereditary spherocytosis**🡪HS or HE or HPP | ***SPTA1*** **NM_003126.3**: c.2898G>A p.(=) het exon 20  ***SPTA1*** **NM_003126.3** Alpha Lely het | Absent | NA | MaxEnt: -29.3% HSF:-10.8% Last base of exon 20 |  | likely pathogenic | -8%  HS  Normal |
| **P23, F, Europe**  **(Healthy daughter P23-1)**  **Congenital dyserythropoiesis? 🡪 X linked sideroblastic anemia?** | ***ALAS2* NM_000032.4*:***  c.-258C>G het promotor | gnomAD All : 0,54% |  |  | Described by Bekri *et al* : Loss of ALAS2 expression  Healthy daughter: absence of the variation. X inactivation study : no inactivation skewing | uncertain significance | Normal  Normal  ND |
| **P24, M, Europe**  **Huge hemolysis with osteonecrosis** 🡪 unknown | ***Negative on CHA genes panel analysis***  ***TRPV4* NM_021625*:*** c.1913C>T (p.P638L) hom exon 13  ***ADAR* NM_001111*:*** c.1586C>T (p.P529L) het exon 2 | gnomAD = 0.03%, no  homozygous recorded  absent | Benign  Disease causing  Tolerated  C0  Probably damaging  Disease causing  Deleterious  C65 | No splicing effect predicted  No splicing effect predicted |  | uncertain significance  likely pathogenic | Normal  ND  ND |
| **P25, M, Maghreb**  **P25-1 : father**  **P25-2 : mother**  **Unexplained hemolysis** 🡪CDAII | ***SEC23B*** **NM_001172745.2**: c.40C>T (p.R14W) het Exon 2/  c.325G>A (p.E109K) het Exon 4 | gnomAD All : 0,022%  / gnomAD All : 0,023% | Possibly damaging  Disease causing  Deleterious  C0/ Probably damaging  Disease causing deleterious  C0 | No splicing effect predicted  / No splicing effect predicted | Family study  Father and mother heterozygous carriers for each variants 🡪 compound heterozygosity confirmed  Russo *et al* 2011 : case report with the same phenotype and genotype | pathogenic/pathogenic | -9%  HS  Atypical profile of band 3 decrease |
| **P26, M, Europe**  **Xerocytosis and iron overload**🡪HUSa and hemochromatosis | ***HAMP* NM_021175.2**  c.49_54del (p.L17_L18del) het Exon 1  ***HFE* NM_000410.3:**  c.845G>A (p.C282Y) het Exon 4  ***CD46* NM_172359:**  c.402T>G (p.I134M) het Exon 4 | Absent  gnomAD All : 3,37%  Absent | NA  Probably damaging  Polymorphism  Deleterious  C65  Possibly damaging  Polymorphism  Deleterious  C0 | No splicing effect predicted  No splicing effect predicted  No splicing effect predicted | *HAMP*: Loss of 2 amino acids in the pre-pro-peptide region in the adressing signal pathway | uncertain significance  pathogenic  uncertain significance | ND  ND  ND |
| **P27**, **F, Europe**  **Neonatal unexplained hemolysis** 🡪unknown  **P27-1 : affected mother**  **P27-2 : affected maternal aunt** | ***CFH NM_00186.3:***  c.2850G>T  (p.Q950H) het Exon 18 | gnomAD All : 0,39% | Benign  Polymorphism  Deleterious  C0 | No splicing effect predicted | Described by Mohlin *et al* in 2015  as a variation with « potentially disease-risk increasing » in atypical HUS  Sanger targeted family study performed in the affected mother and maternal aunt is negative | likely benign | Normal  Normal  ND |
| **P28**, **M, Maghreb**  **Unexplained hemolysis** 🡪unknown | ***SEC23B*** **NM_001172745.2:** c.1276G>A (p.V426I) het exon 11  ***CDAN1* NM_138477:**  c.256C>T (p.P86S) het exon 2 | Described  gnomAD All : 4,3%  gnomAD All 0.052% | Benign  Polymorphism  Tolerated  C0  Benign  Polymorphism  Tolerated  C0 | No splicing effect predicted  No splicing effect predicted | Described by Schwartz *et al* 2009 in a case of CDAII associated with others mutations of *SEC23B* | uncertain significance  uncertain significance | ND  ND  ND |
| **P29, M, Europe**  **Hemolysis +++ on A/S** 🡪HE or HPP | ***SPTA1*** **NM_003126.3**: c.1688G>A (p.R563Q) het exon 14  c.6531-12C>T het Alpha LELY intron 45 | gnomAD All : 0,11% | Probably damaging  Disease causing  Tolerated  C0 | Possible creation of acceptor cryptic site score HSF 83% vs 85%WT (possible truncated protein of 4 AA) |  | uncertain significance | -7%  Atypical  ND |
| **P30, F, Africa**  **Myelodysplasia and**  **Unexplained hemolysis** 🡪 unknown | ***PIEZO1*** **NM_001142864.2**: c.1126C>G (p.P376A) het  Exon 10 | Absent | Benign  Polymorphism  Tolerated  C0 | Possible creation of cryptic acceptor splicing site score HSF 82,7 vs 86,07 for the canonic site of intron 9, possible stop codon premature in exon 10 |  | uncertain significance | Increasing of fluorescence  Atypical : increasing in osmotic resistance without red blood cell dehydration  ND |
| **P31, M, Italy**  **DHSt** 🡪  GARDOS  **P31-1, M, affected son** | ***KCNN4*** **NM_002250.2**: c.1055G>A (p.R352H) het exon 7  ***PIEZO1*** **NM_001142864.2**: c.3629C>T (p.A1210V) het exon 25  ***KCNN4*** **NM_002250.2**: c.1055G>A (p.R352H) het exon 7 | Absent  gnomAD All : 0,006%  Absent | Possibly damaging  Disease causing  Deleterious  C0  Benign  Disease causing  Tolerated  C0  Possibly damaging  Disease causing  Deleterious  C0 | No splicing effect predicted *in silico*  Possible creation of a cryptic site but with score <<to the score of canonic site  No splicing effect predicted *in silico* | Described by Rappetti *Mauss et al* 2015  Described by Rappetti Mauss *et al* 2015 | pathogenic  likely benign  pathogenic | Increasing of fluorescence  Atypical  ND  Normal  Atypical  ND |
| **P32, F, Africa**  **hemolysis in A/S** 🡪unknown | ***SPTB*** **NM_001024858.2**: c.[6706C>A; 6737C>T]  p.[L2236M ; A2246V]  exon 33 | Absent/absent | Benign  Disease causing  Deleterious  C0/  Probably damaging  Polymorphism  Tolerated  C0 | No splicing effect predicted  / No splicing effect |  | uncertain significance/uncertain significance | Normal  Atypical  ND |
| **P33, F, Europe**  hemolytic anemia needing transfusions🡪unknown  **P33-1 : mother**  **P33-2 : father** | ***G6PD* NM_ 000402.4**:  c.538G>A (p.V180I) het exon 5  ***SPTB* NM_001024858.2:** c.6271C>A (p.P2091T) het exon 31  ***G6PD* NM_ 000402.4**:  c.538G>A (p.V180I) het exon 5  ***SPTB* NM_001024858.2:** c.6271C>A (p.P2091T) het exon 31  No mutations in genes panel | Absent/gnomAD All : 0,0065% | possibly damaging  disease causing  Deleterious  C25/Benign  Disease causing  Tolerated  C0 | Inactivation of X chromosome in favor of the X chromosome with the *G6PD* mutation  Mutation of the *SPTB* gene concerns the second base of the exon 31 but no predicted deleterious effect on splicing | Mother presenting hemolysis has the same genotype for *G6PD and SPTB* | likely pathogenic/likely benign | Normal  Normal  ND  ND  Normal  ND |
| **P34, M, Europe**  **Unexplained hemolysis** 🡪 hemochromatosis and susceptibility to HUSa | ***HFE*** NM_000410.3: c.187C>G (p.H63D) hom exon 2  ***ABCG8*** NM_022437: 5’UTR c.-27G>A  ***ADAMTS13*** NM_139025: c.119C>G (p.Ala40Gly) het exon 2/ c.4007G>A (p.R1336Q) het exon 28 | gnomAD : 10.83%  Absent  gnomAD 0,00041%/ gnomAD : 0,0012% | benign  tolerated  Polymorphism  C0  NA  Benign/Deleterious  polymorphism  Tolerated  C0 | No splicing effect predicted | Kaczorowska-Hac *et al* 2016  Activity ADAMTS13 61% : in favor of mutations in cis | uncertain significance  uncertain significance  uncertain significance/ uncertain significance | -5%  ND  ND |
| **P35, M, Europe**  **Unexplained hemolysis** 🡪 myeloproliferative syndrome and susceptibility to pain | Negative on genes panel analysis  ***SCN9A* NM_002977.3**: c.2938G>T (p.A980S**)** het exon 17  ***SH2B3* NM_005475.2:** c.1A>G (p. 0?) het exon 2 | Absent  Absent | Possibly damaging  Disease causing  Deleterious  C0  Probable loss of ATG initiator | No predicted effect on splicing  No predicted effect on splicing |  | uncertain significance  likely pathogenic | -5%  Quasi normal  ND |
| **P36, F, Comoros islands**  **Unexplained hemolysis in A/S** 🡪HE or HPP + SEA ovalocytosis + DHSt + A/S  **P36-1, P36 affected daughter** | ***SPTA1*NM_003126.3*:*** c.6672A>C (p.E2224D) hom exon 48  ***SLC4A1* NM_000342.2*:*** c.1199_1225del (p.A400_A408del) het exon 11  ***PIEZO1*NM_001142864.2*:*** c.1369C>T (p.R457C) het exon 12  *G6PD* NM_000402 : c.292G>A (p.V98M) het exon 4  ***SPTA1*NM_003126.3*:*** c.6672A>C (p.E2224D) het exon 48  ***SLC4A1* NM_000342.2*:*** c.1199_1225del (p.A400_A408del) het exon 11  ***PIEZO1*NM_001142864.2*:*** c.1369C>T (p.R457C) het exon 12  *G6PD* NM_000402 exon 4 : negative | gnomAD Afr = 1.5%  gnomAD homozygous count = 0  SEA Ovalocytosis  gnomAD all : 0,0047%  Absent in gnomAD  gnomAD all : 1.15%  gnomAD Afr = 1.5%  SEA Ovalocytosis  gnomAD all : 0,0047%  Absent in gnomAD | Probably damaging  Disease causing  Deleterious  C35  Probably damaging  Disease causing  Deleterious  C0  Probably damaging  Disease causing  Deleterious  C35  Probably damaging  NA  Tolerated  C0 | No predicted effect on splicing  No predicted effect on splicing  No predicted effect on splicing | Wilder *et al* 2009  Russo *et al* 2018  Vulliamy *et al* 1988  Variant named Matera A- | likely pathogenic  likely pathogenic  likely pathogenic  pathogenic | -23%  ND  ND  -23%  In favor of SEA ovalocytosis  ND |
| **P37, M, Europe**  **TPI deficiency**🡪HPP | ***SPTA1*NM_003126.3*:*** c.3291G>A (p.W1097*) het exon 23  c.6531-12C>T alpha lely het  ***HFE***NM_000410.3: c.187C>G (p.H63D) hom exon 2 | Absent  polymorphism alpha Lely  gnomAD :10.83% | STOP  benign  tolerated  Polymorphism  C0 | Probable mRNA decay  No splicing effect predicted | Kaczorowska-Hac *et al* 2016 | pathogenic  uncertain significance | Normal but after recent transfusion  In favor of erythrocytic membranopathy (context of recent transfusion)  ND |
| **P38, F, Europe**  **unexplained hemolysis** 🡪HUSa + DMLA | ***CFH NM_00186.3***: c.157C>T (p.R53C) het exon 2  ***PIEZO1*NM_001142864.2:** c.4246G>A (p.G1416R) het exon 31 | gnomAD : 0.0014%  gnomAD : 0.0033% | probably damaging  disease causing  deleterious  C0  probably damaging  disease causing  benign  C0 | No predicted effect on splicing  creation of a potential cryptic acceptor site | Fakhouri *et al* 2010  Servais *et al* 2012  R53C decreases inhibitor activity of factor H  No reference | likely pathogenic  uncertain significance | normal  normal  ND |
| **P39, F, Africa**  **unexplained hemolysis HE or HPP**🡪 HPP | ***SPTA1*NM_003126.3***:* c.779T>C (p.L260P) het exon 6  c.6531-12C>T het intron 45 | gnomAD Afr. : 0.017%  polymorphism alpha Lely | probably damaging  disease causing  deleterious  C0 | No predicted effect on splicing | Marchesi S.L *et al* 1987 : decreasing of tetramer spectrin formation due to this mutation, associated to HE | Likely pathogenic  Benign | -7%  Elliptocytosis with probable alpha thal trait  ND |
| **P40, M, Maghreb**  hereditary spherocytosis --> *ATP11C* mutation | ***ATP11C NM_173694:*** c.2434C>T (p.P812S) hem exon 21  ***ANK1 NM_020476:*** *c.4558G>C (p.E1520Q) het exon 38* | gnomAD : 0.0011% (no hemizygous recorded)  gnomAD : 0.0021% | probably damaging  disease causing  tolerated  C0  benign  disease causing  tolerated  C0 | No predicted effect on splicing  No predicted effect on splicing |  | Likely pathogenic  uncertain significance | -5%  atypical HS  ND |

Table S11 Legend: Variants description and classification according to ACMG guidelines as benign, likely benign, uncertain significance, likely pathogenic or pathogenic. *In silico* study of missense variations was assessed thanks to Polyphen-2, Mutation taster, Sift and Align GVGD. HGMD professional and pubmed web interface were used to check for variants description. Abbreviations: het: heterozygous state; hom: homozygous state; hem: hemizygous state; F: female; M: male; HS: hereditary spherocytosis; gnomAD: genome agregation database <https://gnomad.broadinstitute.org> ; ND: not done ; NA: not a

**Supplementary Material and methods**

Sequencing and data analysis

**Library preparation**. Fragmentation of 100 ng of genomic DNA was performed using either mechanical fragmentation with a Bioruptor apparatus (Diagenode Diagnostics, Seraing (Ougrée) - Belgium) or enzymatic fragmentation using Kapa library hyperprep kit following manufacturer recommendations (KAPABIOSYSTEMS, Wilmington, Massachusetts). Then, enrichment for exonic sequences was performed using Medexome kit following manufacturer recommendations (NimbleGen, Madison, Wisconsin).

**Sequencing**. The captured libraries were sequenced on a Nextseq500 instrument (Illumina, [San Diego, California, USA](https://www.google.com/search?client=firefox-b&q=San+Diego&stick=H4sIAAAAAAAAAOPgE-LSz9U3MDIvMUxPUeIAsc0Ny4q0tLKTrfTzi9IT8zKrEksy8_NQOFYZqYkphaWJRSWpRcUANbq9m0QAAAA&sa=X&ved=2ahUKEwilxM7mlqzfAhXNxYUKHRMgDYkQmxMoATAcegQICBAP)) with high-output FlowCell and reagent, in order to obtain 150 bp paired-end reads.

**Bioinformatic analyses**. Demultiplexing and .fastq files generation were performed using Bcl2fastq software (v.1.8). Alignment and variant calling were performed through an in-house pipeline following the BWA/GATK gold standard best practices. The variant calling files (VCF) were annotated using both Annovar and Ingenuity Variant Analysis (Qiagen, Hilden, Germany) on hg19 reference genome assembly and the first intention interpretative analysis was performed based on the pre-established list of 68 CHA genes (Table 1). Another targeted analysis was performed using a different pipeline (Seqnext software, JSI, Ettenheim, Germany) and the same liste of 68 genes. An extended whole-exome analysis was performed as a third line analysis in patients for which CHA origin remained elusive/ was not fully understood, using IVA software filters (phenotypical ranking tools, biological context and inter-patients comparisons). Alamut (Interactive biosoftware, Rouen, France) and IGV (Broad institute, Cambridge, MA 02142, USA) were used as viewers of aligned sequences. Mean depth of coverage was 175x and depth (>30x) was checked using bam files and alamut on regions of interest. Coverage was correct on our genes list expected for exon 1 of *GPX1 and PGD* who were completed by Sanger sequencing when they was analyzed. Exon 1 of the *PIEZO1* gene is covered under 30x (15x) and was not completed by Sanger sequencing.

*In silico* pathogenic prediction was performed on novel missense variations using Polyphen-2 (<http://genetics.bwh.harvard.edu/pph2/>), SIFT (http://sift.jcvi.org/), Mutation Taster (http://www.mutationtaster.org/), align GVGD (<http://agvgd.iarc.fr/>). MaxEntScan (<http://genes.mit.edu/burgelab/maxent/Xmaxentscan_scoreseq.html>) and Human Splicing Finder3.0 (<http://www.umd.be/HSF/>) were used for splicing effect predictions with.

**Open source genomic databases.** The public variation database gnomAD (Broad institute, Cambridge, MA 02142, USA, http://gnomad.broadinstitute.org) was checked for presence and allelic frequency of each genetic variation identified in the 40 patients. We filtered variants according to their frequency in gnomAD. For recessive disorders we only kept variants under 5% of minor allele frequency (MAF) and for autosomic dominant ones those under 1% of MAF.

All new variants have been registered in the Clinvar database (Supplementary table 1)

**Sanger sequencing.** Sanger sequencing was used to confirm each potentially deleterious variation found in the patients. Primers sequences and PCR conditions are available upon request.

Hematologic and Biochemical specialized tests.

**Biochemical and hematologic tests**

In our study we have performed cell blood count, blood smear evaluation, biochemical hemolysis markers dosages, red blood cells density by phthalates gradient determination (with density curves profiles), hemoglobin electrophoresis (to exclude unstable hemoglobins), determination of red blood cells enzymes activity (G6PD, Pyruvate kinase, hexokinase, Pyrimidine 5’nucleotidase) and EMA test for membrane defects. When possible, ektacytometry and protein membrane electrophoresis were performed in another laboratory.

In details, **EMA test** was performed following technology described by King et al in 2000 (26) and Girodon et al 2008 (27). A flow cytometry test using eosin-5’-maleimide (EMA) -labeled red blood cells (RBC) was performed immediately after reception; if not, samples were stored at 4°C 7 days maximum, according to previous published recommendations . The method consists of RBC labeling with the EMA dye and subsequent measurement of the RBC mean fluorescence intensity (MFI) by flow cytometry (26). Samples were always tested in comparison with 6 age-matched controls collected the same day under similar conditions. Red blood cells were washed three times by adding 3 ml of a Dulbecco’s phosphate-buffered saline solution (1X DPBS, 14190-094, Gibco® by life technologies TM) to 1 ml of blood; tubes were centrifuged 5 minutes at 2500 rpm. After plasma withdrawal, 25 µl of the EMA solution diluted at 0.5 mg/ml (63184-10MG, SIGMA®) was added to 5 µl of packed RBCs in capped plastic microtubes, vortexed and incubated 1 hour in the dark at room temperature. RBCs were then washed three times with PBS as described above and resuspended in 2 ml of PBS. MFI was then measured in the FL1 channel of the cytometer (Navios, BeckmanCoulter®); note that before use, the FL1-voltage of the analyzer was standardized against Flow Set Pro fluorospheres (A63492, BeckmanCoulter®) to a predetermined MFI value. The patient’s MFI was further compared to the MFI of the 6 age-matched controls by calculation of a ratio as follows: [(mean of the 6 age-matched controls’ MFI - patient’s MFI)/ mean of the 6 age-matched controls’ MFI] *100). In this study, we used the cut-off value published by Girodon et al. (27)A decreased ratio ≥21%-was positive (and the patient considered having a hereditary abnormality of RBC membrane (mainly hereditary spherocytosis); conversely, a decreased ratio <16% was negative. Values between 16 and 21% (“area of uncertainty”) needed further analysis (ektacytometry, genetic analysis) to confirm or refute the hypothesis of a RBC membrane abnormality

**Density of red blood cells** was measured following the phthalate density-distribution technique as described by Bartolucci *et al* in 2012 (28)**.**

**RNA extraction and transcript analyses** Total RNA was extracted from blood Paxgene tubes, (using Maxwell 16 extraction technology (Promega, Madison, Wisconsin, USA) following manufacturer recommendations. cDNA was generated and then sequenced by Sanger sequencing.

Patients description

Density of red blood cells measured following the phthalate density-distribution technique has been done in 30 patients out 40 with a complete curve.

Two sub-groups have been formed. First one with the 20 suspected patients with hereditary spherocytosis based on principally their positive EMA test results (19 out of 20). The other sub-group is the one of the 20 cases of unexplained hemolysis. It comprises patients with clinico-phenotypical discordance, those with normal or sub-normal EMA test, those with suspicion of CDA and the A/S ones with hemolysis.
